# Intrinsic Dynamic and Static Nature of Halogen Bonding in Neutral Polybromine Clusters, with the Structural Feature Elucidated by QTAIM Dual-Functional Analysis and MO Calculations

**DOI:** 10.3390/molecules26102936

**Published:** 2021-05-14

**Authors:** Satoko Hayashi, Taro Nishide, Eiichiro Tanaka, Waro Nakanishi

**Affiliations:** Faculty of Systems Engineering, Wakayama University, 930 Sakaedani, Wakayama 640-8510, Japan; s209004@wakayama-u.ac.jp (T.N.); s216156@wakayama-u.ac.jp (E.T.)

**Keywords:** ab initio calculations, quantum theory of atoms-in-molecules (QTAIM), bromide, structures

## Abstract

The intrinsic dynamic and static nature of noncovalent Br-∗-Br interactions in neutral polybromine clusters is elucidated for Br_4_–Br_12_, applying QTAIM dual-functional analysis (QTAIM-DFA). The asterisk (∗) emphasizes the existence of the bond critical point (BCP) on the interaction in question. Data from the fully optimized structures correspond to the static nature of the interactions. The intrinsic dynamic nature originates from those of the perturbed structures generated using the coordinates derived from the compliance constants for the interactions and the fully optimized structures. The noncovalent Br-∗-Br interactions in the L-shaped clusters of the *C*_s_ symmetry are predicted to have the typical hydrogen bond nature without covalency, although the first ones in the sequences have the vdW nature. The L-shaped clusters are stabilized by the *n*(Br)→σ*(Br–Br) interactions. The compliance constants for the corresponding noncovalent interactions are strongly correlated to the *E*(2) values based on NBO. Indeed, the MO energies seem not to contribute to stabilizing Br_4_ (*C*_2h_) and Br_4_ (*D*_2d_), but the core potentials stabilize them, relative to the case of 2Br_2_; this is possibly due to the reduced nuclear–electron distances, on average, for the dimers.

## 1. Introduction

Halogen bonding is of current and continuous interest [1,2]. A lot of information relevant to halogen bonding has been accumulated so far [3]. Halogen bonding has been discussed on the basis of the shorter distances between halogen and other atoms in crystals [4,5,6]. The short halogen contacts are found in two types: symmetric (type **I**) and bent (type **II**) geometries. The bonding has also been investigated in the liquid [7,8] and gas [9] phases. The nature of halogen bonding has been discussed based on the theoretical background on the molecular orbital description for the bonding and the σ-hole developed on the halogen atoms, together with the stability of the structural aspects [10]. We also reported the dynamic and static nature of Y–X---π(C_6_H_6_) interactions recently [11]. Halogen bonding is applied to a wide variety of fields in chemical and biological sciences, such as crystal engineering, supramolecular soft matters, and nanoparticles. Efforts have been made to unify and categorize the accumulated results and establish the concept of halogen bonding [3,12,13,14,15].

**Figure 1:**
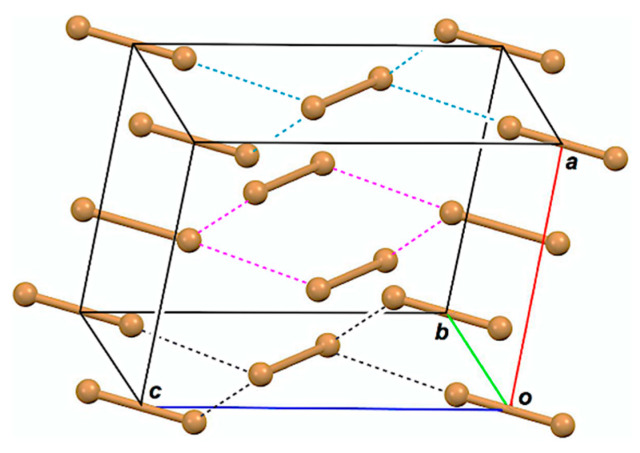
Structure of Br_2_, determined by X-ray crystallographic analysis [17].

Structures of halogen molecules (X_2_) have been reported, as determined by X-ray crystallographic analysis for X = Cl, Br, and I [16,17,18]. The behavior of bromine–bromine interactions has been reported for the optimized structures of Br_2_–Br_5_ in the neutral and/or charged forms, together with Br_1_, so far [19,20]. Figure 1 draws the observed structure of Br_2_, for example. The bromine molecules seem to exist as a zig-zag structure in the infinite chains in crystals. One would find the linear alignment of three Br atoms in an L-shaped dimer ((Br_2_)_2_; Br_4_) and the linear alignment of four Br atoms in a double L-shaped trimer ((Br_2_)_3_; Br_6_) in a planar Br_2_ layer in addition to Br_2_ itself. The linear four Br atoms are located in the two L-shaped dimers of Br_6_, overlapped at the central Br_2_. While the L-shaped dimers seem to construct the zig-zag type infinite chains, the linear four Br atoms construct linear infinite chains. The attractive n_p_(Br)→σ*(Br–Br) σ(3c–4e) (three center–four electron interaction of the σ-type) and n_p_(Br)→σ*(Br–Br)←n_p_(Br) σ(4c–6e) must play a very important role to stabilize Br_4_ and Br_6_, respectively, where n_p_(Br) stands for the p-type nonbonding orbital of Br in the plane, perpendicular to the molecular Br_2_ axis, and σ*(Br–Br) is the σ*-orbital of Br_2_. The crystal structures of Cl_2_ and I_2_ are very similar to that of Br_2_.

We have been very interested in the behavior of halogen bonding in polyhalogen clusters, together with the structures. How can the interactions in the polyhalogen clusters be clarified? We propose QTAIM dual-functional analysis (QTAIM-DFA) [21,22,23,24,25] based on the quantum theory of atoms in molecules (QTAIM) approach introduced by Bader [26,27] to classify and characterize the various interactions effectively [28]. In QTAIM-DFA, *H*_b_(***r***_c_) are plotted versus *H*_b_(***r***_c_) − *V*_b_(***r***_c_)/2 (=(*ћ*^2^/8*m*)∇^2^*ρ*_b_(***r***_c_) (see Equation (SA2) in the supplementary materials), where *ρ*_b_(***r***_c_), *H*_b_(***r***_c_), and *V*_b_(***r***_c_) stand for the charge densities, total electron energy densities, and potential energy densities, respectively, at bond critical points (BCPs, ∗) on the bond paths (BPs) in this paper [26]. The kinetic energy densities at BCPs will be similarly denoted by *G*_b_(***r***_c_) [26]. A chemical bond or an interaction between Br and Br is denoted by Br-∗-Br in this work, where the asterisk emphasizes the existence of a BCP on a BP for Br–Br [26,27]. In our treatment, data from the fully optimized structures are plotted together with those from the perturbed structures around the fully optimized ones. The static nature of the interactions corresponds to the data from the fully optimized structures, which are analyzed using polar coordinate (*R*, *θ*) representation [21,22,23,24,25]. On the other hand, the dynamic nature originates based on the data from both the perturbed and fully optimized structures [21,22,23,24,25]. The plot is expressed by (*θ*_p_, *κ*_p_), where *θ*_p_ corresponds to the tangent line and *κ*_p_ is the curvature of the plot. *θ* and *θ*_p_ are measured from the *y*-axis and the *y*-direction, respectively. We call (*R*, *θ*) and (*θ*_p_, *κ*_p_) the QTAIM-DFA parameters [29].

Interactions are classified by the signs of ∇^2^*ρ*_b_(***r***_c_) and *H*_b_(***r***_c_), based on the QTAIM approach. The interactions are called shard shell (SS) interactions when ∇^2^*ρ*_b_(***r***_c_) < 0 and closed-shell (CS) interactions when ∇^2^*ρ*_b_(***r***_c_) > 0 [26]. In particular, CS interactions are called *pure* CS (*p*-CS) interactions when *H*_b_(***r***_c_) > 0 and ∇^2^*ρ*_b_(***r***_c_) > 0. We call interactions where *H*_b_(***r***_c_) < 0 and ∇^2^*ρ*_b_(***r***_c_) > 0 *regular* CS (*r*-CS) interactions, which clearly distinguishes these interactions from the *p*-CS interactions. The signs of ∇^2^*ρ*_b_(***r***_c_) can be replaced by those of *H*_b_(***r***_c_) − *V*_b_(***r***_c_)/2 because (*ћ*^2^/8*m*)∇^2^*ρ*_b_(***r***_c_) = *H*_b_(***r***_c_) − *V*_b_(***r***_c_)/2 (see Equation (SA2) in the supporting information). Indeed, *H*_b_(***r***_c_) − *V*_b_(***r***_c_)/2 = 0 corresponds to the borderline between the classic covalent bonds of SS and the noncovalent interactions of CS, but *H*_b_(***r***_c_) = 0 appears to be buried in the noncovalent interactions of CS. As a result, it is difficult to characterize the various CS interactions based on the signs of *H*_b_(***r***_c_) − *V*_b_(***r***_c_)/2 and/or *H*_b_(***r***_c_). In QTAIM-DFA, the signs of the first derivatives of *H*_b_(***r***_c_) − *V*_b_(***r***_c_)/2 and *H*_b_(***r***_c_) (d(*H*_b_(***r***_c_) − *V*_b_(***r***_c_)/2)/d*r* and d*H*_b_(***r***_c_)/d*r*, respectively, where *r* is the interaction distance) are used to characterize CS interactions, in addition to those of *H*_b_(***r***_c_) − *V*_b_(***r***_c_)/2 and *H*_b_(***r***_c_), after analysis of the plot. While the former corresponds to (*θ*_p_, *κ*_p_), the latter does to (*R*, *θ*). The analysis of the plots enables us to characterize the various CS interactions more effectively. Again, the details are explained later.

The perturbed structures necessary for QTAIM-DFA can be generated. Among them, a method employing the coordinates corresponding to the compliance constants *C_ii_* for internal vibrations is shown to be highly reliable to generate the perturbed structures [30,31,32,33,34,35,36,37,38,39]. The method, which we proposed recently, is called CIV. The dynamic nature of interactions based on the perturbed structures with CIV is described as the “intrinsic dynamic nature of interactions” since the coordinates are invariant to the choice of coordinate system. Rough criteria that distinguish the interaction in question from others are obtained by applying QTAIM-DFA with CIV to standard interactions. QTAIM-DFA and the criteria are explained in the appendix of the supplementary materials using Schemes SA1–SA3, Figures SA1 and SA2, Table SA1, and Equations (SA1)–(SA7). The basic concept of the QTAIM approach is also explained.

QTAIM-DFA, using the perturbed structures generated with CIV, is well-suited to elucidate the intrinsic dynamic and static nature of halogen–halogen interactions in the polyhalogen clusters. As the first step to clarify the nature of various types of halogen–halogen interactions in the polyhalogen clusters, the nature of each bromine–bromine interaction in the neutral polybromine clusters is elucidated by applying QTAIM-DFA. Various types of structures and interactions are found in the optimized structures of polybromine clusters, other than those observed in the crystals. Here, we present the results of investigations on the polybromine clusters, together with the structural feature, elucidated with QTAIM-DFA and QC calculations.

## 2. Methodological Details in Calculations

The structures were optimized by employing Gaussian 09 programs [40]. The 6-311+G(3df) basis [41,42,43,44] set was applied to optimize the structures of neutral polybromine clusters, Br_2_–Br_12_. The Møller–Plesset second-order energy correlation (MP2) level [45,46,47] was applied for the optimizations. Optimized structures were confirmed by frequency analysis. The results of the frequency analyses were employed to calculate the *C_ij_* values and coordinates corresponding to *C_ii_* [30,34,35,36]. The *ρ*_b_(***r***_c_), *H*_b_(***r***_c_) − *V*_b_(***r***_c_)/2 (=(*ћ*^2^/8*m*)∇^2^*ρ*_b_(***r***_c_)), and *H*_b_(***r***_c_) values were calculated using the Gaussian 09 program package [40], with the same method applied to the optimizations. Data were analyzed with the AIM2000 [48,49] and AIMAll [50] programs.

Coordinates corresponding to the compliance constants for an internal coordinate *i* of the internal vibrations (**C***_i_*) were employed to generate the perturbed structures necessary in QTAIM-DFA [21,22,23,24,25]. Equation (1) explains the method to generate the perturbed structures with CIV. An *i*-th perturbed structure in question (**S***_iw_*) was generated by the addition of the coordinates (**C***_i_*) corresponding to *C_ii_* to the standard orientation of a fully optimized structure (**S**_o_) in the matrix representation. The coefficient *g_iw_* in Equation (1) controls the difference in structures between **S***_iw_* and **S**_o_: *g_iw_* are determined to satisfy Equation (2) for the interaction in question, where *r* and *r*_o_ show the distances in question in the perturbed and fully optimized structures, respectively, with *a*_o_ of Bohr radius (0.52918 Å) [21,22,23,24,25,30].

**S***_iw_* = **S**_o_ + *g_iw_* × **C***_i_* (1)

*r* = *r*_o_ + *wa*_o_ (*w* = (0), ±0.05 and ±0.1; *a*_o_ = 0.52918 Å) (2)

*y* = *c*_o_ + *c*_1_*x* + *c*_2_*x*^2^ + *c*_3_*x*^3^   (*R*_c_^2^: square of correlation coefficient) (3)

In the QTAIM-DFA treatment, *H*_b_(***r***_c_) are plotted versus *H*_b_(***r***_c_) − *V*_b_(***r***_c_)/2 for the data of five points of *w* = 0, ±0.05, and ±0.1 in Equation (2). Each plot is analyzed using a regression curve of the cubic function, as shown in Equation (3), where (*x*, *y*) = (*H*_b_(***r***_c_) − *V*_b_(***r***_c_)/2, *H*_b_(***r***_c_)) (*R*_c_^2^ (square of correlation coefficient) > 0.99999 in the norm) [25].

## 3. Results and Discussion

### 3.1. Structural Optimizations of Polybromine Clusters, Br_6_–Br_12_

Structures of the neutral Br_2_–Br_12_ clusters were optimized with MP2/6-311+G(3df). The structural parameters for the optimized structures of minima for Br_2_–Br_6_ and Br_8_–Br_12_ are collected in Tables S1 and S2, respectively. Some transition states (TSs) for Br_4_ and Br_6_ were also calclaterd. The notation of *C*_s_-L*_m_* (*m* = 1–5) is used for the linear L-shaped clusters of the *C*_s_ symmetry, where *m* stands for the number of noncovalent interactions in Br_2*m*+2_ (*m* = 1–5). Cyclic structures are also optimized, retaining the higher symmetries. The optimized structures are not shown in figures, but they can be found in the molecular graphs with the contour maps of *ρ*(***r***) for the linear-type bromine clusters Br_4_–Br_12_ (*C*_s_-L*_m_* (*m* = 1–5)) and for the cyclic bromine clusters Br_4_–Br_12_, drawn on the optimized structures with MP2/6-311+G(3df) [51]. The energies for the formation of Br_4_–Br_6_ and Br_8_–Br_12_ are given in Tables S1 and S2, respectively, from the components (∆*E* = *E*(Br_2*k*_) − *kE*(Br_2_)) on the energy surfaces (∆*E*_ES_) and those with the collections of zero-point energies (∆*E*_ZP_). The ∆*E*_ZP_ values were plotted versus ∆*E*_ES_. The plot is shown in Figure S1, which gives an excellent correlation (*y* = 0.940*x* + 0.129; *R*_c_^2^ (square of correlation coefficient) = 0.9999) [52]. Therefore, the ∆*E*_ES_ values are employed for the discussion.

The behavior of the neutral dibromine clusters (Br_4_) is discussed first. Three structures were optimized for Br_4_ as minima with some TSs. The minima are the L-shaped structure of *C*_s_ symmetry (Br_4_ (*C*_s_-L_1_)) [19], the cyclic structure of *C*_2h_ symmetry (Br_4_ (*C*_2h_)), and the tetrahedral type of *D*_2d_ symmetry (Br_4_ (*D*_2d_)). A TS of the *C*_s_ symmetry was detected between Br_4_ (*C*_s_-L_1_) and Br_4_ (*C*_2h_), and two TSs of the *C*_1_ symmetry were between Br_4_ (*C*_2h_) and Br_4_ (*D*_2d_) and between Br_4_ (*D*_2d_) and Br_4_ (*C*_s_-L_1_). They are called TS (*C*_s_: *C*_s_, *C*_2h_), TS (*C*_1_: *C*_2h_, *D*_2d_), and TS (*C*_1_: *D*_2d_, *C*_s_), respectively. The three minima will be converted to each other through the three TSs. A TS between Br_4_ (*C*_s_-L_1_) and its topological isomer was also detected, which is called TS (*C*_2v_: *C*_s_, *C*_s_); however, further effort was not made to search for similar TSs between Br_4_ (*C*_2h_) and its topological isomer and between Br_4_ (*C*_2d_) and its topological isomer.

**Figure 2:**
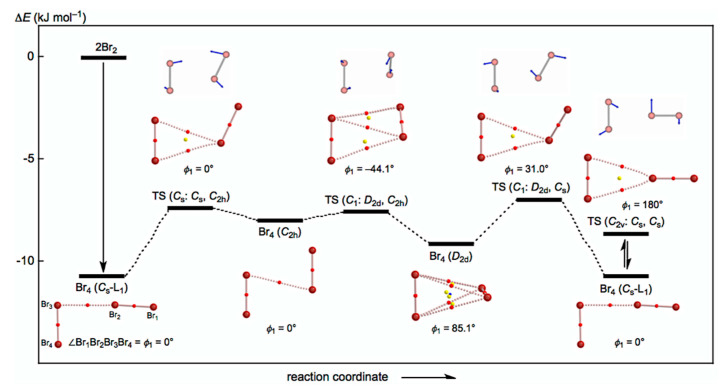
Energy profile with molecular graphs for the structures of Br_4_ clusters, optimized with MP2/6-311+G(3df).

Figure 2 draws the energy profiles for the optimized structures of minima, Br_4_ (*C*_s_-L_1_), Br_4_ (*C*_2h_), and Br_4_ (*D*_2d_), together with the TSs TS (*C*_s_: *C*_s_, *C*_2h_), TS (*C*_s_: *C*_2h_, *D*_2d_), TS (*C*_1_: *C*_2d_, *C*_s_), and TS (*C*_2v_: *C*_s_, *C*_s_). The optimized structures are not shown in the figures, but they can be found in the molecular graphs shown in Figure 2, illustrated on the optimized structures. All BCPs expected are detected clearly, together with RCPs and a CCP [26]. The Δ*E*_ES_ value of −10.7 kJ mol^−1^ for the formation of Br_4_ (*C*_s_-L_1_) seems very close to the border area between the vdW and typical hydrogen bond (*t*-HB) adducts. The driving force for the formation of Br_4_ (*C*_s_-L_1_) must be Br_3_ σ(3c–4e) of the *n*_p_(Br)→σ*(Br–Br) type. The interactions in Br_4_ (*C*_2h_) and Br_4_ (*D*_2d_) seem very different from those in Br_4_ (*C*_s_-L_1_). The Δ*E*_ES_ values of Br_4_ (*C*_2h_) (−8.0 kJ mol^−1^) and Br_4_ (*D*_2d_) (−9.1 kJ mol^−1^) are close to that for Br_4_ (*C*_s_-L_1_) (−10.7 kJ mol^−1^). Moreover, the values for TS (*C*_s_: *C*_s_, *C*_2h_) (−7.4 kJ mol^−1^), TS (*C*_1_: *C*_2h_, *D*_2d_) (−7.6 kJ mol^−1^), TS (*C*_1_: *D*_2d_, *C*_s_) (−7.0 kJ mol^−1^), and TS (*C*_2v_: *C*_s_, *C*_s_) (−8.7 kJ mol^−1^) are not so different from those for the minima.

In the case of Br_6_, three structures of the linear *C*_s_ symmetry (Br_6_ (*C*_s_-L_2_)), the linear *C*_2_ symmetry (Br_6_ (*C*_2_)), and the cyclic *C*_3h_ symmetry (Br_6_ (*C*_3h_-*c*)) were optimized typically as minima. The linear Br_6_ clusters of *C*_2h_ symmetry (Br_6_ (*C*_2h_)) and *C*_2v_ symmetry (Br_6_ (*C*_2v_)), similar to Br_6_ (*C*_2_), were also optimized, of which the torsional angles, *ϕ*(^1^Br^2^Br^5^Br^6^Br) (=*ϕ*_3_), were 0° and 180°, respectively. One imaginary frequency was detected for each; therefore, they are assigned to TSs between Br_6_ (*C*_2_) and the topological isomer on the different reaction coordinates. Further effort was not made to search for TSs.

The Δ*E*_ES_ value for Br_6_ (*C*_s_-L_2_) was predicted to be −22.6 kJ mol^−1^. The magnitude is slightly larger than the double value for Br_4_ (*C*_s_-L_1_) (∆*E*_ES_ = −10.7 kJ mol^−1^). Two types of σ (3c–4e) operate to stabilize Br_6_ (*C*_s_-L_2_). One, σ(3c–4e), seems similar to that in Br_4_ (*C*_s_-L_1_), but the other would be somewhat different. Namely, the second interaction would contribute to ∆*E*_ES_ somewhat more than that of the first one in the formation of Br_6_ (*C*_s_-L_2_). On the other hand, the linear interaction in Br_6_ (*C*_2_) can be explained by σ(4c–6e) of the *n*_p_(Br)→σ*(Br–Br)←*n*_p_(Br) type. The magnitude of ∆*E*_ES_ of Br_6_ (*C*_2_) seems slightly smaller than that of Br_6_ (*C*_s_-L_2_) but is very close to the double value for Br_4_ (*C*_s_-L_1_). The magnitude of ∆*E*_ES_ for Br_6_ (*C*_3h_-*c*) is close to the triple value of Br_4_ (*C*_s_-L_1_). One finds triply degenerated σ(3c–4e) interactions in Br_6_ (*C*_3h_-*c*). The similarity in the interactions for Br_4_ (*C*_s_-L_1_), Br_6_ (*C*_2_), and Br_6_ (*C*_3h_-*c*) will be discussed again later. The magnitudes of ∆*E*_ES_ become proportionally larger to the size of the clusters, as shown in Figures S1 and S2. The Δ*E*_ES_ values are plotted versus *k* in Br_2*k*_ (2 ≤ *k* ≤ 6) for the *C*_s_-L*_m_* type. The results are shown in Figure S2. Contributions from inner σ(3c–4e) (named *r*_in_) to Δ*E*_ES_ seem slightly larger than those from σ(3c–4e) in the front end and end positions (named *r*_2_ and *r*_ω_, respectively).

After examination of the optimized structures, the next extension is to clarify the nature of Br-∗-Br interactions by applying QTAIM-DFA. The contour plots are discussed next.

### 3.2. Molecular Graphs with Contour Plots of Polybromine Clusters

**Figure 3:**
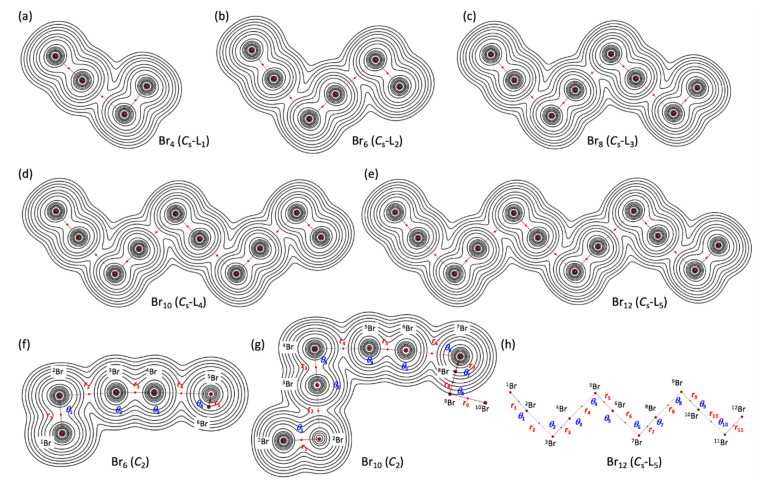
Molecular graphs with contour plots of *ρ*(***r***) for the linear-type bromine clusters of Br_4_–Br_12_, calculated with MP2/6-311+G(3df). (**a**–**e**) for the linear *C*_s_-L*_m_* type, (**f**,**g**) for the *C*_2_ type, and (**h**) for the notations of atoms, bonds, and angles, exemplified by B_12_ (*C*_s_-L_5_). BCPs are denoted by red dots, and BPs (bond paths) are by pink lines. Bromine atoms are in reddish-brown.

**Figure 4:**
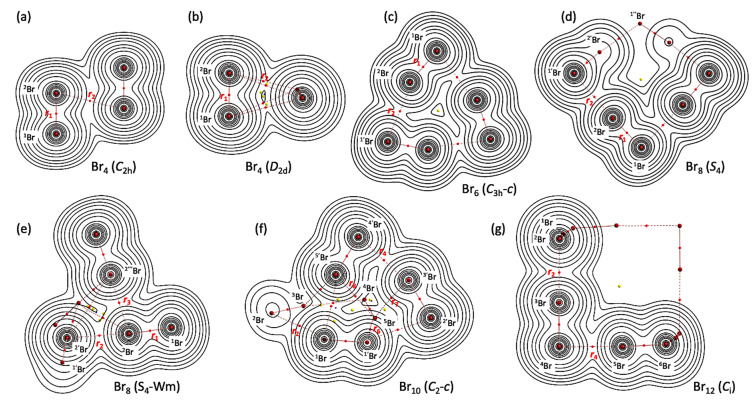
Molecular graphs with contour plots of *ρ*(***r***) for the cyclic bromine clusters of Br_4_–Br_12_, (**a**–**g**), calculated with MP2/6-311+G(3df). BCPs are denoted by red dots, RCPs (ring-critical points) by yellow dots, CCPs (cage-critical points) by blue dots, and BPs (bond paths) by pink lines. See ref. [55] for (**a**).

Figure 3 illustrates the molecular graphs with contour maps of *ρ*(***r***) for the linear type of Br_4_ (*C*_s_-L_1_)–Br_12_ (*C*_s_-L_5_), drawn on the structures optimized with MP2/6-311+G(3df). Figure 4 draws the molecular graphs with contour maps of *ρ*(***r***) for Br_4_–Br_12_, other than those for Br_4_ (*C*_s_-L_1_)–Br_12_ (*C*_s_-L_5_), calculated with MP2/6-311+G(3df) [53,54] (see also Figure S3). All BCPs expected are detected clearly, together with RCPs and a CCP containing those for noncovalent Br-∗-Br interactions, which are located at the (three-dimensional) saddle points of *ρ*(***r***).

### 3.3. Survey of the Br-∗-Br Interactions in Polybromine Clusters

As shown in Figure 2, Figure 3 and Figure 4, the BPs in Br_4_–Br_12_ seem almost straight. The linearity is confirmed by comparing the lengths of BPs (*r*_BP_) with the corresponding straight-line distances (*R*_SL_). The *r*_BP_ and *R*_SL_ values are collected in Table S3, together with the differences between them, Δ*r*_BP_ (=*r*_BP_ − *R*_SL_). The magnitudes of Δ*r*_BP_ are less than 0.01 Å, except for *r*_2_ in Br_4_ (*C*_2v_) (Δ*r*_BP_ = 0.014 Å), *r*_3_ in Br_8_ (*S*_4_-Wm) (0.014 Å), and *r*_2_ in Br_10_ (*C*_2_-*c*) (0.012 Å). Consequently, all BPs in Br_4_–Br_12_ can be approximated as straight lines.

**Figure 5:**
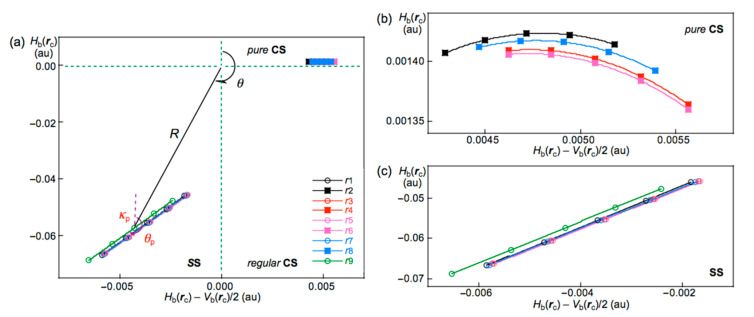
QTAIM-DFA plots (*H*_b_(***r***_c_) versus *H*_b_(***r***_c_) − *V*_b_(***r***_c_)/2) for the interactions in Br_10_ (*C*_s_-L_4_), evaluated with MP2/6-311+G(3df); (**a**) whole region, (**b**) *pure* CS region, and (**c**) SS region. Marks and colors are shown in the figure.

The *ρ*_b_(***r***_c_), *H*_b_(***r***_c_) − *V*_b_(***r***_c_)/2 (=(*ћ*^2^/8*m*)∇^2^*ρ*_b_(***r***_c_)), and *H*_b_(***r***_c_) values are calculated for the Br-∗-Br interactions at BCPs in the structures of Br_2_–Br_12_, optimized with MP2/6-311+G(3df) [53,54,55]. Table 1 collects the values for the noncovalent Br-∗-Br interactions in Br_4_–Br_12_ of the *C*_s_-L*_m_* type. Table 2 summarizes the values for the noncovalent Br-∗-Br interactions in Br_4_–Br_12_, other than those of the *C*_s_-L*_m_* type. *H*_b_(***r***_c_) are plotted versus *H*_b_(***r***_c_) − *V*_b_(***r***_c_)/2 for the data shown in Table 1 and Table 2, together with those from the perturbed structures generated with CIV. Figure 5 shows the plots for the noncovalent Br-∗-Br interactions and covalent Br-∗-Br bonds, exemplified by Br_10_ (*C*_s_-L_4_).

QTAIM-DFA parameters of (*R*, *θ*) and (*θ*_p_, *κ*_p_) are obtained by analyzing the plots of *H*_b_(***r***_c_) versus *H*_b_(***r***_c_) − *V*_b_(***r***_c_)/2, according to Equations (S3)–(S6). Table 1 collects the QTAIM-DFA parameters for the noncovalent Br-∗-Br interactions of Br_4_ (*C*_s_-L_1_)–Br_12_ (*C*_s_-L_5_), Br_6_ (*C*_2_), and Br_10_ (*C*_2_) together with the *C_ii_* values. Table 2 collects the (*R*, *θ*) and (*θ*_p_, *κ*_p_) values for Br_4_–Br_12_, other than those given in Table 1, together with the *C_ii_* values. The (*R*, *θ*) and (*θ*_p_, *κ*_p_) values for the covalent Br-∗-Br bonds in Br_4_–Br_12_ are collected in Table S4.

### 3.4. The Nature of Br-∗-Br Interactions in Polybromine Clusters

The nature of the covalent and noncovalent Br-∗-Br interactions in Br_2_–Br_12_ is discussed on the basis of the (*R*, *θ*, *θ*_p_) values, employing standard values as a reference (see Scheme SA3).

It is instructive to survey the criteria shown in Scheme SA3 before detailed discussion. The criteria tell us that 180° < *θ* (*H*_b_(***r***_c_) − *V*_b_(***r***_c_)/2 < 0) for the SS interactions and *θ* < 180° (*H*_b_(***r***_c_) − *V*_b_(***r***_c_)/2 > 0) for the CS interactions. The CS interactions are subdivided into *pure* CS interactions (*p*-CS) of 45° < *θ* < 90° (*H*_b_(***r***_c_) > 0) and *regular* CS interactions (*r*-CS) of 90° < *θ* < 180° (*H*_b_(***r***_c_) < 0). The *θ*_p_ value predicts the character of interactions. In the *pure* CS region of 45° < *θ* < 90°, the character of interactions will be the vdW type for 45° < *θ*_p_ < 90° and the *typical*-HB type with no covalency (*t*-HB_nc_) for 90° < *θ*_p_ < 125°, where *θ*_p_ = 125° approximately corresponds to *θ* = 90°. The classical chemical covalent bonds of SS (180° < θ) will be strong when *R* > 0.15 au (Cov-s: strong covalent bonds), whereas they will be weak for *R* < 0.15 au (Cov-w: weak covalent bonds).

The (*R*, *θ*, *θ*_p_) values are (0.0576 au, 184.3°, 190.9°) for the original Br_2_ if evaluated with MP2/6-311+G(3df). Therefore, the nature of the Br-∗-Br bond in Br_2_ is classified by the SS interactions (*θ* > 180°) and characterized to have a Cov-w nature (*θ*_p_ > 180° and *R* < 0.15 au). The nature is denoted by SS/Cov-w. The (*R*, *θ*, *θ*_p_) values for the covalent Br-∗-Br bonds in Br_4_–Br_12_ are (0.0472–0.0578 au, 182.0–184.4°, 190.4–192.1°); therefore, their nature is predicted to be SS/Cov-w. The nature of the covalent Br-∗-Br bonds seems unchanged in the formation of the clusters [53,54]. The noncovalent Br-∗-Br interactions in Br_4_–Br_12_ are all classified by *pure* CS interactions since *θ* ≤ 76° (<< 90°) [53,54]. The *θ*_p_ values in the *C*_s_-L*_m_* clusters change systematically. The *θ*_p_ values for *r*_2_ in Br_2*k*_ (*C*_s_-L*_m_*) (*k* = 2–6) are predicted to be in the range of 89.1° ≤ *θ*_p_ ≤ 89.6°, with *θ*_p_ = 87.9° for Br_4_ (*C*_s_-L_1_).

However, the values for *r_n_*_-2_ in Br_2*k*_ (*C*_s_-L*_m_*) (*k* = 2–6) are in the range of 90.6° ≤ *θ*_p_ ≤ 91.2° and the values for noncovalent interactions, other than edge positions, are in the range of 92.1° ≤ *θ*_p_ ≤ 93.0°. Namely, the noncovalent Br-∗-Br interactions are predicted to have the vdW nature (*p*-CS/vdW) for *r*_2_, while the interactions other than *r*_2_ are predicted to have the *t*-HB_nc_ nature (*p*-CS/*t*-HB_nc_) since *θ*_p_ > 90°. The *θ*_p_ values of *r*_2_ for the *C*_s_-L*_m_* clusters will be less than 90°, irrespective of the angles between *r*_1_ and *r*_2_, which are close to 180°. The *θ*_p_ values will be larger than 90° for all noncovalent interactions other than *r*_2_. Table 1 contains the data for Br_10_ (*C*_2_), of which *θ*_p_ = 90.4° (> 90°) for *r*_2_ and *θ*_p_ = 87.1° (<90°) for *r*_4_, although Br_10_ (*C*_2_) is not the *C*_s_-L*_m_* type. The results for *r*_2_ seem reasonable based on the structure (cf. Figure 3), while those for *r*_4_ would be complex. Table 1 summarizes the predicted nature.

In the case of the noncovalent Br-∗-Br interactions in Br_4_–Br_12_, other than the *C*_s_-L*_m_* type clusters, *θ*_p_ > 90° for *r*_2_ in Br_8_ (*S*_4_) (*θ*_p_ = 93.4°) and Br_8_ (*S*_4_-Wm) (*θ*_p_ = 94.8°) and for *r*_2_, *r*_4_, and *r*_6_ in Br_12_ (*C*_i_) (93.4° ≤ *θ*_p_ ≤ 93.7°). The interactions would have the *t*-HB_nc_ nature (*p*-CS/*t*-HB_nc_). Very weak noncovalent Br-∗-Br interactions are also detected. The ranges of 64.2° ≤ *θ* ≤ 66.6° and 66.2° ≤ *θ*_p_ ≤ 71.2° are predicted for *r*_2_ and *r*_3_ in Br_4_ (*C*_2h_), *r*_2_ in Br_4_ (*C*_2v_), *r*_3_ in Br_8_ (*S*_4_-Wm), and *r*_7_ and *r*_8_ in Br_10_ (*C*_2_-*c*). The results are summarized in Table 2.

What are the relationships between the QTAIM-DFA parameters for the noncovalent Br-∗-Br interactions? The *θ* and *θ*_p_ values are plotted versus *R*. The plots are shown in Figure S4; they give very good correlations. The *θ*_p_ values are plotted versus *θ*. The plot is shown in Figure S5; it also gives a very good correlation. Table 3 summarizes the correlations among the QTAIM-DFA parameters.

To further examine the behavior of noncovalent Br-∗-Br interactions, NBO analysis is applied to the interactions.

### 3.5. NBO Analysis for Br-∗-Br of Br_4_ (C_s_-L_1_)–Br_12_ (C_s_-L_5_)

The noncovalent Br-∗-Br interactions in Br_4_(*C*_s_-L_1_)–Br_12_ (*C*_s_-L_5_) are characterized by σ(3c–4e) of the *n*(Br)→σ*(Br–Br) type. NBO analysis [56] was applied to the *n*(Br)→σ*(Br–Br) interactions with MP2/6-311+G(3df). For each donor NBO (*i*) and acceptor NBO (*j*), the stabilization energy *E*(2) is calculated based on the second-order perturbation theory in NBO. The *E*(2) values are calculated according to Equation (4), where *q_i_* is the donor orbital occupancy, *ε_i_*, *ε_j_* are diagonal elements (orbital energies), and *F*(*i,j*) is the off-diagonal NBO Fock matrix element. The values are obtained separately by the contributions from *n*_s_(Br)→σ*(Br–Br) and *n*_p_(Br)→σ*(Br–Br), which are summarized in Table S5. The total values corresponding to *n*_s+p_(Br)→σ*(Br–Br) (=*n*_s_(Br)→σ*(Br–Br) + *n*_p_(Br)→σ*(Br–Br)) were calculated, which are also summarized in Table S5. The total values are employed for the discussion.

*E*(2) = *q_i_* × *F*(*i,j*)^2^/(*ε_j_* − *ε_i_*) (4)

**Figure 6:**
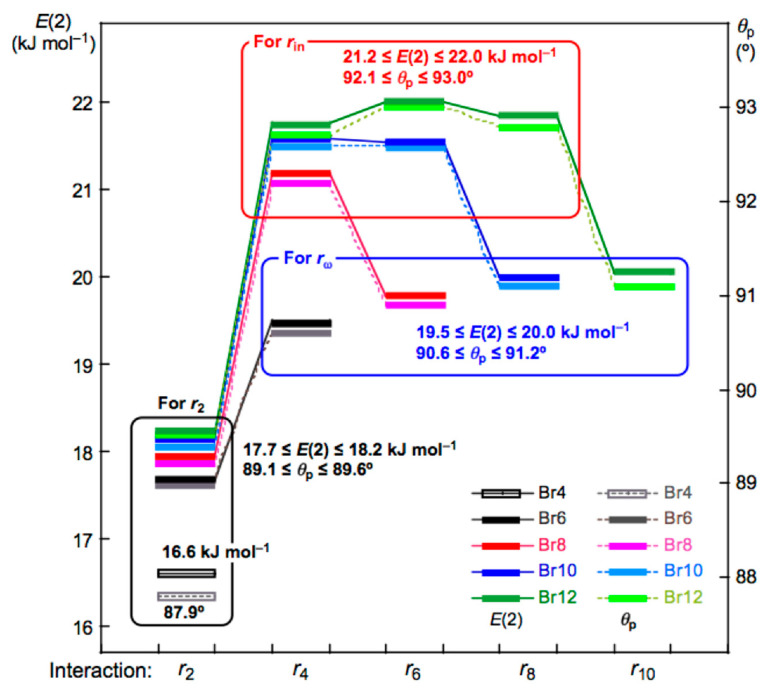
Plots of *θ*_p_ and *E*(2) for the noncovalent Br-∗-Br interactions in Br_4_ (*C*_s_-L_1_)–Br_12_ (*C*_s_-L_5_). Colors are shown in the figure.

Figure 6 shows the plots of *E*(2) and *θ*_p_ for the noncovalent Br-∗-Br interactions in Br_4_ (*C*_s_-L_1_)–Br_12_ (*C*_s_-L_5_). The values become larger in the order of P (*r*_2_: Br_4_ (*C*_s_-L_1_)) < P (*r*_2_: Br_6_ (*C*_s_-L_2_)–Br_12_ (*C*_s_-L_5_)) < P (*r*_ω_: Br_6_ (*C*_s_-L_2_)–Br_12_ (*C*_s_-L_5_)) < P (*r*_in_: Br_6_ (*C*_s_-L_2_)–Br_12_ (*C*_s_-L_5_)), where P means *E*(2) or *θ*_p_, while *r*_ω_ and *r*_in_ stand for the last end and the inside noncovalent interactions, respectively, in the sequence (see Figure 2 and Figure 3). The values for P = *E*(2) are as follows: *E*(2) = 16.6 kJ mol^−1^ for *r*_2_ in Br_4_ (*C*_s_-L_1_) < 17.7 ≤ *E*(2) ≤ 18.2 kJ mol^−1^ for *r*_2_ in Br_6_ (*C*_s_-L_2_)–Br_12_ (*C*_s_-L_5_) < 19.5 ≤ *E*(2) ≤ 20.0 kJ mol^−1^ for *r*_ω_ in Br_6_ (*C*_s_-L_2_)– Br_12_ (*C*_s_-L_5_) < 21.2 ≤ *E*(2) ≤ 22.0 kJ mol^−1^ for *r*_in_ in Br_8_ (*C*_s_-L_3_)–Br_12_ (*C*_s_-L_5_).

**Figure 7:**
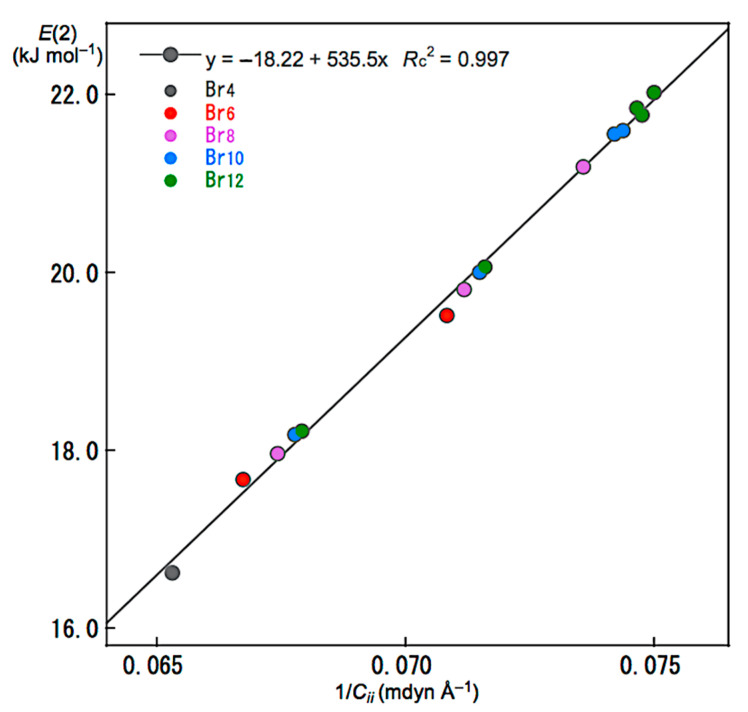
Plot of *E*(2) versus 1/*C_ii_* for the noncovalent Br-∗-Br interactions in Br_4_ (*C*_s_-L_1_)–Br_12_ (*C*_s_-L_5_).

Relations between *E*(2) and *C_ii_* were also examined for noncovalent Br-∗-Br interactions in Br_4_ (*C*_s_-L_1_)–Br_12_ (*C*_s_-L_5_). The *E*(2) values were plotted versus *C_ii_*^−1^ for the noncovalent interactions. Figure 7 shows the plot. The plot gives a very good correlation, which is shown in Table 3 (Entry 5). The results show that the energies for σ(3c–4e) of the *n*_p_(Br)→σ*(Br–Br) type in Br_4_ (*C*_s_-L_1_)–Br_12_ (*C*_s_-L_5_) are well evaluated, not only by *E*(2) but also by *C_ii_*^−1^. Similar relations would be essentially observed for the interactions in the nonlinear clusters; however, the analyses will be much complex due to the unsuitable structures for the NBO analysis, such as the deviations in the interaction angles expected for Br_3_ σ(3c–4e), the mutual interactions between Br_3_ σ(3c–4e), and/or the steric effect from other bonds and interactions, placed proximity in space. The *E*(2) values for Br_4_ (*C*_s_-L_1_)–Br_12_ (*C*_s_-L_5_) were also plotted versus *R*, *θ*, and *θ*_p_, shown in Figures S6–S8, respectively. The plots give very good correlations, which are given in Table 3 (Entries 6–8).

### 3.6. MO Descriptions for Noncovalent Br-∗-Br Interactions in Br_4_

As discussed above, Br_3_ σ(3c–4e) of the *n*_p_(Br)→σ*(Br–Br) type plays an important role in the formation of Br_4_ (*C*_s_-L_1_)–Br_12_ (*C*_s_-L_5_). However, there must exist some interactions, other than Br_3_ σ(3c–4e), to stabilize the clusters. The Δ*E*_ES_ values for Br_4_ (*C*_2h_) (−8.0 kJ mol^−1^) and Br_4_ (*D*_2d_) (−9.1 kJ mol^−1^) are not so different from that for Br_4_ (*C*_s_-L_1_) (−10.7 kJ mol^−1^). However, Br_4_ (*C*_2h_) and Br_4_ (*D*_2d_) must consist of interactions other than σ(3c–4e). Indeed, Br_3_ σ(3c–4e) of the *n*(Br)→σ*(Br–Br) type contributes to stabilizing Br_4_ (*C*_s_-L_1_), but Br_4_ (*C*_2h_) and Br_4_ (*D*_2d_) are shown to be stabilized by the σ(Br–Br)→σ*Ry(Br) interaction by NBO, where Ry stands for the Rydberg term, although not shown.

The total energy for a species (*E*) is given by the sum of the core terms (*H_c_*(*i*)) over all electrons, Σ*_i_^n^ H_c_*(*i*), and the electron–electron repulsive terms, (Σ*_i_*_≠*j*_*^n^ J_ij_* − Σ*_i_*_≠*j*,‖_*^n^ K_ij_*)/2, as shown by Equation (5), where *H_c_*(*i*) consists of the kinetic energy and electron–nuclear attractive terms for electron *i*. *E* contains the nuclear–nuclear repulsive terms, although not clearly shown in Equation (5). As shown in Equation (6), the sum of MO energy for electron *i*, *ε_i_*, over all electrons, Σ*_i_*_=1_*^n^ ε_i_*, will be larger than *E* by (Σ*_i_*_≠*j*_*^n^ J_ij_* − Σ*_i_*_≠*j*,‖_*^n^ K_ij_*)/2 since the electron–electron repulsions are doubly counted in Equation (6). Therefore, Σ*_i_^n^ H_c_*(*i*) and (Σ*_i_*_≠*j*_*^n^ J_ij_* − Σ*_i_*_≠*j*,‖_*^n^ K_ij_*)/2 are given separately by Equations (7) and (8), respectively. The *ε_i_* values for Br_4_ (*C*_2h_), Br_4_ (*D*_2d_), and 2Br_2_, together with Br_4_ (*C*_s_-L_1_), are collected in Tables S6–S9, respectively, for convenience of discussion. Parameters (Δ*P*) in the formation of Br_2*k*_ from the components are evaluated according to Equation (9). The ΔΣ*_i_^n^ H_c_*(*i*) and Δ(Σ*_i_*_≠*j*_*^n^ J_ij_* − Σ*_i_*_≠*j*,‖_*^n^ K_ij_*)/2 values for the formation of Br_4_ (*C*_2h_), Br_4_ (*D*_2d_), and Br_4_ (*C*_s_-L_1_) are collected in Table S11.

*E* = Σ*_i_^n^ H_c_*(*i*) + (Σ*_i_*_≠*j*_*^n^ J_ij_* − Σ*_i_*_≠*j*,‖_*^n^ K_ij_*)/2 (5)

Σ_*i*=1_^*n*^*ε_i_* = Σ*_i_^n^H_c_*(*i*) + (Σ_*i*≠*j*_^*n*^*J_ij_* − Σ_*i*≠*j*,‖_^*n*^*K_ij_*) (6)

Σ_*i*_^*n*^*H_c_*(*i*) = 2*E* − Σ_*i*=1_^*n*^*ε_i_* (7)

(Σ_*i*≠*j*_^*n*^*J_ij_* − Σ_*i*≠*j*,‖_^*n*^*K_ij_*)/2 = Σ_*i*=1_^*n*^*ε_i_* − *E* (8)

Δ*P*(Br_2*k*_) = *P*(Br_2*k*_) − *kP*(Br_2_) (9)

The nature of noncovalent Br---Br interactions in Br_4_ (*C*_s_-L_1_) is examined first. The σ(3c–4e) character in Br_4_ (*C*_s_-L_1_) is confirmed by the natural charge evaluated with NPA (*Qn*), developed in the formation of Br_4_ (*C*_s_-L_1_). The evaluated *Qn* values are Br(1: −0.0128|e^−^|)–Br(2: −0.0002|e^−^|)---Br(3: −0.0010|e^−^|)–Br(4: 0.0140|e^−^|); therefore, *Qn*(Br(4)–Br(3)) and *Qn*(Br(2)–Br(1)) are +0.013|e^−^| and −0.013|e^−^|, respectively. Each MO in Br_4_ (*C*_s_-L_1_) is almost localized on Br(4)–Br(3) or Br(2)–Br(1), except for a few cases. MOs in Br_4_ (*C*_s_-L_1_) must be affected by the local charge. Each MO energy in Br_4_ (*C*_s_-L_1_) seems higher than the corresponding value of 2Br_2_ by 10–20 kJ mol^−1^ if the MO is localized on Br(2)–Br(1), lower by 15–25 kJ mol^−1^ on Br(3)–Br(4), and slightly lower by 0–5 kJ mol^−1^ if the MO is localized on the whole molecule. We should be careful since it depends on the phase in MO and the position of the Br atom(s). Typical cases are shown in Figure S9. In total, ΔΣ*_i_*_=1_*^n^* *ε_i_* is evaluated to be −357.2 kJ mol^−1^ for Br_4_ (*C*_s_-L_1_). The results show that Br_4_ (*C*_s_-L_1_) is stabilized in the formation of the dimer from the components through the lowering of MO energies in total, which is consistent with those evaluated with NBO, as discussed above.

**Figure 8:**
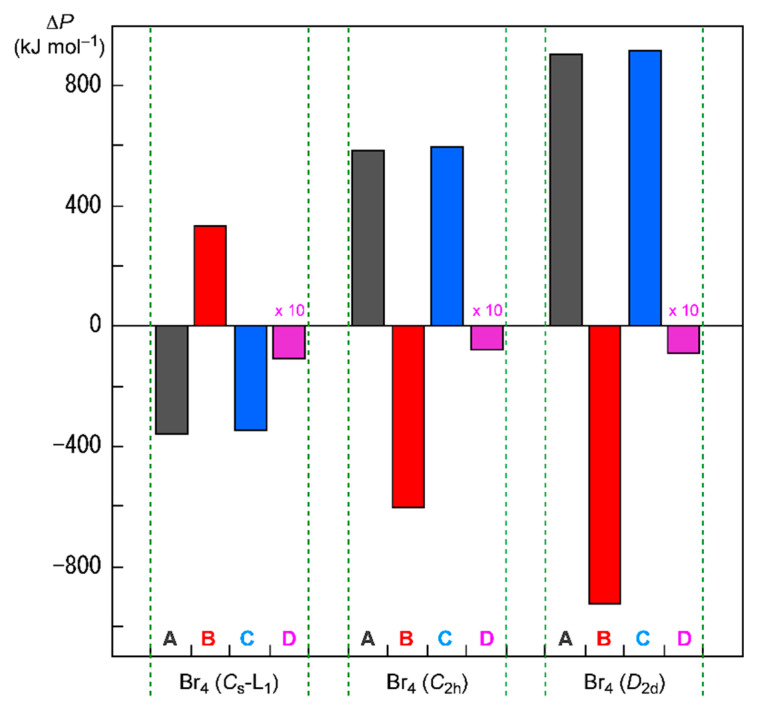
Contributions from ΔΣ*_i_^n^H_c_*(*i*) (=Δ*P* = **B**) and Δ(Σ*_i_*_≠*j*_*^n^ J_ij_* − Σ*_i_*_≠*j,*‖_*^n^ K_ij_*)/2 (=Δ*P* = **C**) to Δ*E*_ES_ (=Δ*P* = **D**, magnified by 10 times in the plot) for Br_4_ (*C*_s_-L_1_), Br_4_ (*C*_2h_), and Br_4_ (*D*_2d_), relative to 2Br_2_, together with ΔΣ*_i_*_=1_*^n^* *ε_i_* (=Δ*P* = **A**).

Figure 8 shows the plots of ΔΣ*_i_^n^ H_c_*(*i*) and Δ(Σ*_i_*_≠*j*_*^n^ J_ij_* − Σ*_i_*_≠*j,*‖_*^n^ K_ij_*)/2 for Br_4_ (*C*_s_-L_1_), Br_4_ (*C*_2h_), and Br_4_ (*D*_2d_), together with Δ*E*_ES_ and ΔΣ*_i_*_=1_*^n^* *ε_i_*. In the case of Br_4_ (*C*_s_-L_1_), ΔΣ*_i_^n^ H_c_*(*i*) and Δ(Σ*_i_*_≠*j*_*^n^ J_ij_* − Σ*_i_*_≠*j,*‖_*^n^ K_ij_*)/2 are evaluated to be 335.7 and −346.4 kJ mol^−1^, respectively, which stabilizes Br_4_ (*C*_s_-L_1_) in total. Two Br_2_ molecules in Br_4_ (*C*_s_-L_1_) will supply a wider area for electrons without severe disadvantageous steric compression by the L-shaped structure in a plane. The structural feature of Br_4_ (*C*_s_-L_1_) may reduce (or may not severely increase) the electron–electron repulsive terms, Δ((Σ*_i_*_≠*j*_*^n^ J_ij_* − Σ*_i_*_≠*j,*‖_*^n^ K_ij_*)/2), relative to the case of 2Br_2_, although ΔΣ*_i_^n^ H_c_*(*i*) seems to destabilize it. The ΔΣ*_i_^n^ H_c_*(*i*) + Δ(Σ*_i_*_≠*j*_*^n^ J_ij_* − Σ*_i_*_≠*j,*‖_*^n^ K_ij_*)/2 value is equal to −10.7 kJ mol^−1^, which corresponds to the stabilization energy of Br_4_ (*C*_s_-L_1_), relative to 2Br_2_.

The energy profiles of Br_4_ (*C*_2h_) and Br_4_ (*D*_2d_) seem very different from that of Br_4_ (*C*_s_-L_1_). The ΔΣ*_i_*_=1_*^n^* *ε_i_* terms for Br_4_ (*C*_2h_) and Br_4_ (*D*_2d_) are evaluated to be 587.5 and 908.1 kJ mol^−1^, respectively. Namely, Br_4_ (*C*_2h_) and Br_4_ (*D*_2d_) would be less stable than 2Br_2_ if ΔΣ*_i_*_=1_*^n^* *ε_i_* are compared. Consequently, it is difficult to explain the stability of Br_4_ (*C*_2h_) and Br_4_ (*D*_2d_), based on the MO energies. On the other hand, ΔΣ*_i_^n^ H_c_*(*i*) of Br_4_ (*C*_2h_) and Br_4_ (*D*_2d_) are evaluated to be −603.5 and −926.3 kJ mol^−1^, respectively, whereas Δ(Σ*_i_*_≠*j*_*^n^ J_ij_* − Σ*_i_*_≠*j,*‖_*^n^ K_ij_*)/2 are 595.5 and 917.2 kJ mol^−1^, respectively. As a result, the (ΔΣ*_i_^n^ H_c_*(*i*) + Δ(Σ*_i_*_≠*j*_*^n^ J_ij_* − Σ*_i_*_≠*j,*‖_*^n^ K_ij_*)/2) values are −8.0 and −9.1 kJ mol^−1^ for Br_4_ (*C*_2h_) and Br_4_ (*D*_2d_), respectively, which correspond to their Δ*E*_ES_ values (relative to 2*E*(Br_2_)). The results show that the stabilizing effect of ΔΣ*_i_^n^ H_c_*(*i*) overcomes the shorter electron–nuclear distances in the species on average. The shorter electron–electron distances must destabilize Br_4_ (*C*_2h_) and Br_4_ (*D*_2d_) through the factor of Δ(Σ*_i_*_≠*j*_*^n^ J_ij_* − Σ*_i_*_≠*j,*‖_*^n^ K_ij_*)/2, which is the inverse effect from the electron–nuclear interaction on ΔΣ*_i_^n^ H_c_*(*i*). However, the effect of the shorter distances on ΔΣ*_i_^n^ H_c_*(*i*) seems to contribute more effectively than the case of Δ(Σ*_i_*_≠*j*_*^n^ J_ij_* − Σ*_i_*_≠*j,*‖_*^n^ K_ij_*)/2 in Br_4_ (*C*_2h_) and Br_4_ (*D*_2d_), although they are not so large.

**Figure 9:**
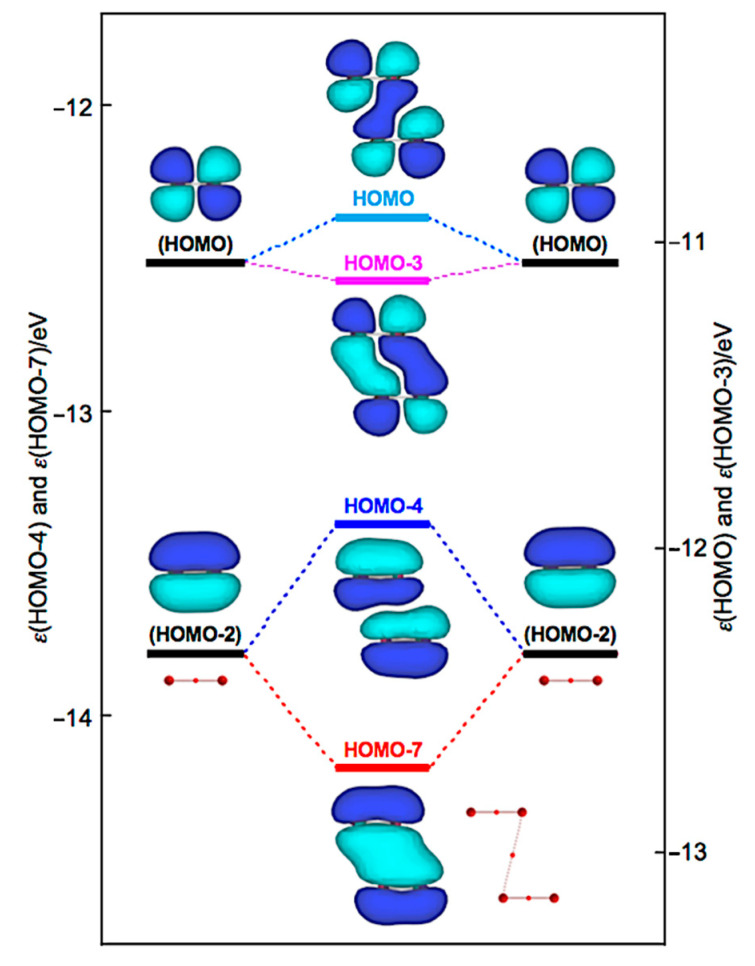
Energy profile for the formation of Br_4_ (*C*_2h_), exemplified by HOMO, HOMO-3, HOMO-4, and HOMO-7.

How can the BPs in Br_4_ (*C*_2h_) and Br_4_ (*D*_2d_) be rationalized through orbital interactions? The Δ*ε_i_* values of Br_4_ (*C*_2h_) are positive for all occupied MOs, relative to the corresponding values of 2Br_2_, except for HOMO-3 (−5.5 kJ mol^−1^), HOMO-6 (−2.9 kJ mol^−1^), HOMO-7 (−35.8 kJ mol^−1^), and HOMO-13 (−1.1 kJ mol^−1^). Figure 9 illustrates the interactions to produce HOMO, HOMO-3, HOMO-4, and HOMO-7. Indeed, HOMO-7 seems to contribute well to stabilizing Br_4_ (*C*_2h_), but HOMO-4 (+40.8 kJ mol^−1^) is also formed in the π(Br_2_)–π(Br_2_) mode. Similarly, HOMO (+13.7 kJ mol^−1^) is formed, together with HOMO-3 in the π*(Br_2_) + π*(Br_2_) mode. Therefore, all MOs seem not to contribute to stabilizing Br_4_ (*C*_2h_) inherently. Nevertheless, HOMO, HOMO-4, and HOMO-7 seem to rationalize the appearance of BPs in Br_4_ (*C*_2h_), along the diagonal line and shorter sides of the parallelogram, although all electrons contribute to the appearance of BPs in molecules.

**Figure 10:**
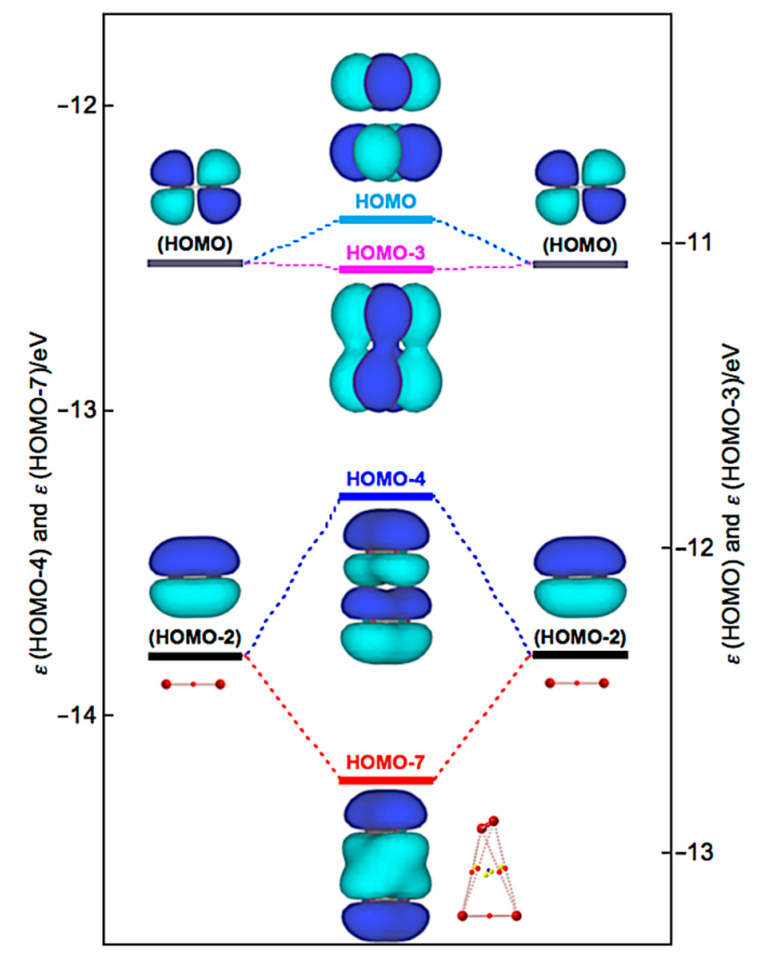
Energy profile for the formation of Br_4_ (*D*_2d_), exemplified by HOMO, HOMO-3, HOMO-4, and HOMO-7.

Similarly, Δ*ε_i_* of Br_4_ (*D*_2d_) are positive for all occupied MOs, relative to the corresponding values of 2Br_2_, except for HOMO-3 (−1.9 kJ mol^−1^), HOMO-7 (−39.2 kJ mol^−1^), and HOMO-13 (−0.5 kJ mol^−1^). Figure 10 illustrates the interactions to produce HOMO, HOMO-3, HOMO-4, and HOMO-7 in Br_4_ (*D*_2d_). HOMO-4 (+50.2 kJ mol^−1^) is formed through the π(Br_2_)–π(Br_2_) mode in addition to HOMO-7. Similarly, HOMO (+13.9 kJ mol^−1^) is formed, accompanied by HOMO-3, in the π*(Br_2_) + π*(Br_2_) mode. Therefore, no MOs essentially stabilize Br_4_ (*D*_2d_). However, the appearance of BPs along the longer and shorter diagonal lines of the tetrahedron of Br_4_ (*D*_2d_) seem to be rationalized by HOMO-7, together with HOMO-3 and HOMO-4, modifying the BPs, although BPs will appear as the whole properties of molecules.

The nature of interactions in the charged clusters is also of interest. Such investigations are in progress.

## 4. Conclusions

The intrinsic dynamic and static nature of noncovalent Br-∗-Br interactions was elucidated for Br_4_–Br_10_ with MP2/6-311+G(3df). QTAIM-DFA was applied to the investigation. *H*_b_(***r***_c_) were plotted versus *H*_b_(***r***_c_) − *V*_b_(***r***_c_)/2 for the interactions at BCPs of the fully optimized structures, together with those from the perturbed structures, generated with CIV. The nature of the covalent Br-∗-Br bonds in Br_4_–Br_10_ is predicted to have the SS/Cov-w nature if calculated with MP2/6-311+G(3df). On the other hand, the nature of the noncovalent Br-∗-Br interactions in Br_4_–Br_12_ is classified by the *pure* CS interactions (*θ* ≤ 76°). The noncovalent Br-∗-Br interactions in the linear type clusters of Br_4_ (*C*_s_-L_1_)–Br_12_ (*C*_s_-L_5_) are predicted to have the *p*-CS/*t*-HB_nc_ nature (90.6° ≤ *θ*_p_), except for *r*_2_, outside the ones of the first end, which have the *p*-CS/vdW nature, although it is very close to the border area between the two (*θ*_p_ ≤ 89.4°). In the case of the cyclic clusters, the noncovalent Br-∗-Br interactions will have the *p*-CS/vdW nature (*θ*_p_ ≤ 88.4°), except for *r*_2_ in Br_8_ (*S*_4_) (*θ*_p_ = 93.5°) and Br_8_ (*S*_4_-Wm) (*θ*_p_ = 95.3°), which have the *p*-CS/*t*-HB_nc_ nature.

The energies for Br_3_ σ(3c–4e) of the *n*_p_(Br)→σ*(Br–Br) type are well evaluated by not only *E*(2) but also *C_ii_*^−1^ for Br_4_ (*C*_s_-L_1_)–Br_12_ (*C*_s_-L_5_). *E*(2) correlates very well to *C_ii_*^−1^. The CT interactions of the *n*_p_(Br)→σ*(Br–Br) type must contribute to form Br_4_ (*C*_s_-L_1_), which can be explained based on the MO energies, *ε_i_*. However, it seems difficult to explain the stability of Br_4_ (*C*_2h_) and Br_4_ (*D*_2d_) based on the energies. The Br_2_ molecules must be stacked more effectively in Br_4_ (*C*_2h_) and Br_4_ (*D*_2d_), resulting in shorter electronuclear distances on average. The energy lowering effect by ΔΣ*_i_^n^ H_c_*(*i*), due to the effective stacking of 2Br_2_ in Br_4_ (*C*_2h_) and Br_4_ (*D*_2d_), contributes to form the clusters, although the inverse contribution from Δ((Σ*_i_*_≠*j*_*^n^ J_ij_* − Σ*_i_*_≠*j,*‖_*^n^ K_ij_*)/2) must also be considered.

## Figures and Tables

**Table 1:** The *ρ*_b_(***r***_c_), *H*_b_(***r***_c_) − *V*_b_(***r***_c_)/2 (=(*ћ*^2^/8*m*)∇^2^*ρ*_b_(***r***_c_)), and *H*_b_(***r***_c_) values and QTAIM-DFA parameters for Br-∗-Br at BCPs in Br_4_ (*C*_s_-L_1_)–Br_12_ (*C*_s_-L_5_), together with Br_10_ (*C*_2_) and Br_2_, evaluated with MP2/6-311+G(3df) ^1^.

**Species**	**BCP on**	***ρ*_b_(*r*_c_)**		***c*∇^2^*ρ*_b_(*r*_c_) ^2^**	***H*_b_(*r*_c_)**	***R*^3^**		***θ*^4^**
(Symmetry)		(au)		(au)	(au)	(au)		(°)
Br_4_ (*C*_s_-L_1_)	*r*_2_	0.0109		0.0045	0.0014	0.0048		72.5
Br_6_ (*C*_s_-L_2_)	*r*_2_	0.0113		0.0047	0.0014	0.0049		73.0
Br_6_ (*C*_s_-L_2_)	*r*_4_	0.0119		0.0049	0.0014	0.0051		73.7
Br_8_ (*C*_s_-L_3_)	*r*_2_	0.0114		0.0047	0.0014	0.0049		73.2
Br_8_ (*C*_s_-L_3_)	*r*_4_	0.0124		0.0050	0.0014	0.0052		74.4
Br_8_ (*C*_s_-L_3_)	*r*_6_	0.0120		0.0049	0.0014	0.0051		73.9
Br_10_ (*C*_s_-L_4_)	*r*_2_	0.0114		0.0047	0.0014	0.0049		73.2
Br_10_ (*C*_s_-L_4_)	*r*_4_	0.0125		0.0051	0.0014	0.0053		74.6
Br_10_ (*C*_s_-L_4_)	*r*_6_	0.0125		0.0051	0.0014	0.0053		74.6
Br_10_ (*C*_s_-L_4_)	*r*_8_	0.0120		0.0049	0.0014	0.0051		73.9
Br_12_ (*C*_s_-L_5_)	*r*_2_	0.0114		0.0047	0.0014	0.0049		73.2
Br_12_ (*C*_s_-L_5_)	*r*_4_	0.0126		0.0051	0.0014	0.0053		74.7
Br_12_ (*C*_s_-L_5_)	*r*_6_	0.0127		0.0051	0.0014	0.0053		74.7
Br_12_ (*C*_s_-L_5_)	*r*_8_	0.0126		0.0051	0.0014	0.0053		74.7
Br_12_ (*C*_s_-L_5_)	*r*_10_	0.0120		0.0049	0.0014	0.0051		73.9
Br_6_ (*C*_2_)	*r*_2_	0.0104		0.0044	0.0014	0.0046		72.1
Br_10_ (*C*_2_)	*r*_2_	0.0118		0.0048	0.0014	0.0050		73.6
Br_10_ (*C*_2_)	*r*_4_	0.0106		0.0044	0.0014	0.0046		72.3
**Species**	***C_ii_*^5^**		***θ*_p:CIV_^6^**		***κ*_p:CIV_^7^**		**Predicted**	
(Symmetry)	(Å mdyn^−1^)		(°)		(au^−1^)		nature	
Br_4_ (*C*_s_-L_1_)	15.311		87.8		121.2		*p*-CS/vdW ^8^	
Br_6_ (*C*_s_-L_2_)	14.984		89.0		124.9		*p*-CS/vdW ^8^	
Br_6_ (*C*_s_-L_2_)	14.114		90.6		127.3		*p*-CS/*t*-HB ^9^	
Br_8_ (*C*_s_-L_3_)	14.826		89.2		125.0		*p*-CS/vdW ^8^	
Br_8_ (*C*_s_-L_3_)	13.590		92.2		132.0		*p*-CS/*t*-HB ^9^	
Br_8_ (*C*_s_-L_3_)	14.048		90.9		127.1		*p*-CS/*t*-HB ^9^	
Br_10_ (*C*_s_-L_4_)	14.751		89.4		126.2		*p*-CS/vdW ^8^	
Br_10_ (*C*_s_-L_4_)	13.445		92.6		133.2		*p*-CS/*t*-HB ^9^	
Br_10_ (*C*_s_-L_4_)	13.478		92.6		132.5		*p*-CS/*t*-HB ^9^	
Br_10_ (*C*_s_-L_4_)	13.983		91.1		128.4		*p*-CS/*t*-HB ^9^	
Br_12_ (*C*_s_-L_5_)	14.719		89.5		126.9		*p*-CS/vdW ^8^	
Br_12_ (*C*_s_-L_5_)	13.376		92.7		133.3		*p*-CS/*t*-HB ^9^	
Br_12_ (*C*_s_-L_5_)	13.334		93.0		134.3		*p*-CS/*t*-HB ^9^	
Br_12_ (*C*_s_-L_5_)	13.393		92.8		132.6		*p*-CS/*t*-HB ^9^	
Br_12_ (*C*_s_-L_5_)	13.962		91.1		128.8		*p*-CS/*t*-HB ^9^	
Br_6_ (*C*_2_)	16.025		86.7		119.2		*p*-CS/vdW ^8^	
Br_10_ (*C*_2_)	14.218		90.2		126.7		*p*-CS/*t*-HB ^9^	
Br_10_ (*C*_2_)	16.378		87.2		120.0		*p*-CS/vdW ^8^	

^1^ The interactions in minima are shown. ^2^ *c*∇^2^*ρ*_b_(***r***_c_) = *H*_b_(***r***_c_) − *V*_b_(***r***_c_)/2, where *c* = *ħ*^2^/8*m*. ^3^ *R* = [(*H*_b_(***r***_c_) − *V*_b_(***r***_c_)/2)^2^ + *H*_b_(***r***_c_)^2^]^1/2^. ^4^ *θ* = 90° − tan^−1^[*H*_b_(***r***_c_)/(*H*_b_(***r***_c_) − *V*_b_(***r***_c_)/2)]. ^5^ Defined in Equation (R1) in the text. ^6^ *θ*_p_ = 90° − tan^−1^(d*y*/d*x*), where (*x*, *y*) = (*H*_b_(***r***_c_) − *V*_b_(***r***_c_)/2, *H*_b_(***r***_c_)). ^7^ *κ*_p_ = |d^2^*y*/d*x*^2^|/[1 + (d*y*/d*x*)^2^]^3/2^. ^8^ The *pure* CS interaction of the vdW nature. ^9^ The *pure* CS interaction of the HB nature without covalency.

**Table 2:** The *ρ*_b_(***r***_c_), *H*_b_(***r***_c_) − *V*_b_(***r***_c_)/2 (=(*ћ*^2^/8*m*)∇^2^*ρ*_b_(***r***_c_)), and *H*_b_(***r***_c_) values and QTAIM-DFA parameters for Br-∗-Br at BCPs in Br_4_–Br_12_, other than the *C*_s_-L*_m_* structures, evaluated with MP2/6-311+G(3df) ^1^.

**Species**	**BCP on**	***ρ*_b_(*r*_c_)**		***c*∇^2^** ***ρ*_b_(*r*_c_) ^2^**	***H*_b_(*r*_c_)**	***R*^3^**		***θ*^4^**
(Symmetry)		(au)		(au)	(au)	(au)		(°)
Br_4_ (*C*_2h_)	*r*_2_	0.0055		0.0022	0.0009	0.0024		67.2
Br_4_ (*D*_2d_)	*r*_2_	0.0042		0.0017	0.0007	0.0018		66.0
Br_6_ (*C*_3h_-*c*)	*r*_2_	0.0092		0.0038	0.0013	0.0040		70.7
Br_8_ (*S*_4_)	*r*_2_	0.0128		0.0051	0.0014	0.0053		74.8
Br_8_ (*S*_4_-Wm) ^5^	*r*_2_	0.0136		0.0054	0.0013	0.0056		76.0
Br_8_ (*S*_4_-Wm) ^5^	*r*_3_	0.0038		0.0015	0.0007	0.0016		66.0
Br_10_ (*C*_2_-*c*)	*r*_2_	0.0087		0.0035	0.0012	0.0037		70.5
Br_10_ (*C*_2_-*c*)	*r*_4_	0.0097		0.0040	0.0014	0.0042		71.3
Br_10_ (*C*_2_-*c*)	*r*_6_	0.0110		0.0044	0.0014	0.0046		73.0
Br_10_ (*C*_2_-*c*)	*r*_7_	0.0049		0.0019	0.0008	0.0021		66.2
Br_10_ (*C*_2_-*c*)	*r*_8_	0.0049		0.0018	0.0008	0.0020		66.6
Br_12_ (*C*_i_)	*r*_2_	0.0129		0.0052	0.0014	0.0054		75.0
Br_12_ (*C*_i_)	*r*_4_	0.0129		0.0052	0.0014	0.0054		75.0
**Species**	***C_ii_*^6^**		***θ*_p:CIV_^7^**		***κ*_p:CIV_^8^**		**Predicted**	
(Symmetry)	(Å mdyn^−1^)		(°)		(au^−1^)		nature	
Br_4_ (*C*_2h_)	24.709		73.6		122.9		*p*-CS/vdW ^9^	
Br_4_ (*D*_2d_)	40.402		69.6		136.3		*p*-CS/vdW ^9^	
Br_6_ (*C*_3h_-*c*)	25.617		83.3		121.7		*p*-CS/vdW ^9^	
Br_8_ (*S*_4_)	13.201		93.5		139.2		*p*-CS/*t*-HB ^10^	
Br_8_ (*S*_4_-Wm) ^5^	11.294		95.3		139.0		*p*-CS/*t*-HB ^10^	
Br_8_ (*S*_4_-Wm) ^5^	52.918		67.5		204.0		*p*-CS/vdW ^9^	
Br_10_ (*C*_2_-*c*)	34.402		81.3		112.7		*p*-CS/vdW ^9^	
Br_10_ (*C*_2_-*c*)	23.971		84.7		122.1		*p*-CS/vdW ^9^	
Br_10_ (*C*_2_-*c*)	20.831		87.6		122.6		*p*-CS/vdW ^9^	
Br_10_ (*C*_2_-*c*)	29.570		71.5		118.9		*p*-CS/vdW ^9^	
Br_10_ (*C*_2_-*c*)	37.855		71.8		120.4		*p*-CS/vdW ^9^	
Br_12_ (*C*_i_)	13.483		93.7		137.9		*p*-CS/*t*-HB ^10^	
Br_12_ (*C*_i_)	13.482		93.7		137.3		*p*-CS/*t*-HB ^10^	

^1^ The interactions in minima are shown. ^2^ c∇^2^*ρ*_b_(***r***_c_) = *H*_b_(***r***_c_) − *V*_b_(***r***_c_)/2, where *c* = *ħ*^2^/8*m*. ^3^ *R* = [*H*_b_(***r***_c_) − *V*_b_(***r***_c_)/2)^2^ + *H*_b_(***r***_c_)^2^]1/2. ^4^ *θ* = 90° − tan^−1^[*H*_b_(***r***_c_)/(*H*_b_(***r***_c_) − *V*_b_(***r***_c_)/2)]. ^5^ Image from windmill. ^6^ Defined in Equation (R1) in the text. ^7^ *θ*_p_ = 90° − tan^−1^(d*y*/d*x*), where (*x*, *y*) = (*H*_b_(***r***_c_) − *V*_b_(***r***_c_)/2, *H*_b_(***r***_c_)). ^8^ *κ*_p_ = |d^2^*y*/d*x*^2^|/[1 + (d*y*/d*x*)^2^]^3/2^. ^9^ The *pure* CS interaction of the vdW nature. ^10^ The *pure* CS interaction of the HB nature without covalency.

**Table 3:** Correlations in the plots ^1^.

Entry	Correlation	*a*	*b*	*R*_c_^2^	*n*
1	Δ*E*_ZP_ vs. Δ*E*_ES_	0.940	0.129	0.9999	20 ^2^
2	*θ* vs. *R*	2595.6	60.70	0.979	33
3	*θ*_p_ vs. *R*	6449.1	58.19	0.989	33
4	*θ*_p_ vs. *θ*	2.67	−106.26	0.992	31 ^3^
5	*E*(2) vs. *C_ii_*^−1^	535.5	−18.22	0.997	15 ^4^
6	*E*(2)*vs*. *R*	9760.9	−29.92	0.983	15 ^4^
7	*E*(2) vs *θ*	2.446	−160.88	0.996	15 ^4^
8	*E*(2) vs. *θ*_p_	1.067	77.17	0.999	15 ^4^

^1^ The constants (*a*, *b*, *R*_c_^2^) are the correlation constant, the *y*-intercept, and the square of the correlation coefficient, respectively, in *y* = *ax* + *b*. ^2^ Containing TS species. ^3^ Neglecting the data of *r*_2_ and *r*_3_ in Br_4_ (*C*_2h_). ^4^ For the noncovalent Br-∗-Br interactions in Br_4_ (*C*_s_-L_1_)–Br_12_ (*C*_s_-L_5_).

## Data Availability

Data are contained within the article or Supplementary Materials.

## Supplementary Materials

The following are available online, Table S1: Structural parameters for Br_2_–Br_6_, Table S2: Structural parameters for Br_8_–Br_12_, Table S3: The bond path distances and the straight-line distances in the polybromide clusters, together with the differences between the two, Table S4: The *ρ*_b_(***r***_c_), *H*_b_(***r***_c_) − *V*_b_(***r***_c_)/2 (=(*ћ*^2^/8*m*)∇^2^*ρ*_b_(***r***_c_)), and *H*_b_(***r***_c_) values and QTAIM-DFA parameters for Br-∗-Br in polybromine clusters of Br_2_–Br_12_, Table S5: Contributions from the donor–acceptor (NBO(*i*)→NBO(*j*)) interactions of the n(Br)→σ*(Br–Br) type in the optimized structures of Br_4_–Br_12_, calculated using NBO analysis, Table S6: MO energies of Br_4_ (*C*_2h_), Table S7: MO energies of Br_4_ (*D*_2d_), Table S8: MO energies of Br_2_ (*D*_∞h_), Table S9: MO energies of Br_4_ (*C*_s_-L_1_), Table S10: The Δ*ε**_i_* values for Br_4_ (*C*_s_-L_1_), relative to 2Br_2_ (*D*_∞h_), Table S11: Energies for the Br_4_ clusters and 2Br_2_, together with the differences between the two, Figure S1: Plot of Δ*E*_ZP_ versus Δ*E*_ES_ for Br_4_–Br_12_, relative to those of Br_2_, respectively, Figure S2: Plots of Δ*E*_ES_ for Br_2_–Br_12_ (*C*_s_-L*_n_*), Figure S3: Optimized structures for the cyclic bromine clusters of Br_8_–Br_12_, together with the linear type bromine cluster of Br_10_, Figure S4: Plot of *θ* and *θ*_p_ versus *R* for the noncovalent Br-∗-Br interactions at the BCPs in the fully optimized structures of Br_4_–Br_12_, Figure S5: Plot of *θ*_p_ versus *θ* for the noncovalent Br-∗-Br interactions at the BCPs in the fully optimized structures of Br_4_–Br_12_, Figure S6: Plot of *E*(2) versus *R* for noncovalent Br-∗-Br interactions in Br_4_ (*C*_s_-L_1_)–Br_12_ (*C*_s_-L_5_), Figure S7: Plot of *E*(2) versus *θ* for noncovalent Br-∗-Br interactions in Br_4_ (*C*_s_-L_1_)–Br_12_ (*C*_s_-L_5_), Figure S8: Plot of *E*(2) versus *θ*_p_ for noncovalent Br-∗-Br interactions in Br_4_ (*C*_s_-L_1_)–Br_12_ (*C*_s_-L_5_), Figure S9: MO*_i_* (*i* = 70, 67, 64, 35, and 30) and the energies relative to those corresponding to 2Br_2_, and Cartesian coordinates and energies of all the species involved in the present work. Appendix: Survey of QTAIM, closely related to QTAIM dual-functional analysis; Criteria for classification of interactions: behavior of typical interactions elucidated by QTAIM-DFA; Characterization of interactions.
