# Supplementary material for: Intrinsic Dynamic and Static Nature of Halogen Bonding in Neutral Polybromine Clusters, with the Structural Feature Elucidated by QTAIM Dual-Functional Analysis and MO Calculations
Source: Molecules. 2021 May 14;26(10):2936. doi: 10.3390/molecules26102936 (PMC8157170; doi:10.3390/molecules26102936)
Supplement: Supplementary file 1 [file molecules-26-02936-s001.zip › molecules-1220542-supplementary.pdf]

Electronic Supplementary Information

Intrinsic dynamic and static nature of halogen bonding in neutral polybromine clusters with the structural feature, elucidated by QTAIM dual functional analysis and MO calculations†

Satoko Hayashi,* Taro Nishide, Eiichiro Tanaka,[a] and Waro Nakanishi*,[a]

Faculty of Systems Engineering, Wakayama University, 930 Sakaedani, Wakayama 640-8510, Japan.
E-mail: nakanisi@sys.wakayama-u.ac.jp, hayashi3@sys.wakayama-u.ac.jp

Table of Contents **Pages**

Table S1. Structural parameters for Br_2–Br_6 2
Table S2. Structural parameters for Br_8–Br_{12} 3
Table S3. The bond path distances and the straight-line distances in the polybromide clusters, together with the differences between the two 4–5
Table S4. The $\rho_b(\boldsymbol{r}_c)$, $H_b(\boldsymbol{r}_c) - V_b(\boldsymbol{r}_c)/2$ (= $(\hbar^2/8m)\nabla^2\rho_b(\boldsymbol{r}_c)$), and $H_b(\boldsymbol{r}_c)$ values and QTAIM-DFA parameters for Br-∗-Br in polybromine clusters of Br_2–Br_{12} 6
Table S5. Contributions from the donor-acceptor (NBO(i)→NBO(j)) interactions of the n(Br)→σ*(Br–Br) type in the optimized structures of Br_4–Br_{12} calculated with the NBO analysis 7
Table S6. MO energies of Br_4 (C_{2h}) 8
Table S7. MO energies of Br_4 (D_{2d}) 9
Table S8. MO energies of Br_2 ($D_{\infty h}$) 9
Table S9. MO energies of Br_4 (C_s-L_1) 8
Table S10. The $\Delta\varepsilon_i$ values for Br_4 (C_s-L_1), relative to $2Br_2$ ($D_{\infty h}$) 10
Table S11. Energies for the Br_4 clusters and $2Br_2$, together with the differences between the two 10
Figure S1. Plot of ΔE_{ZP} versus ΔE_{ES} for Br_4–Br_{12}, relative to those of Br_2, respectively 11
Figure S2. Plots of ΔE_{ES} for Br_2–Br_{12} (C_s-L_n) 11
Figure S3. Optimized structures for the cyclic bromine clusters of Br_8–Br_{12}, together with the linear type bromine cluster of Br_{10} 12
Figure S4. Plot of θ and θ_p versus R for the non-covalent Br-∗-Br interactions at the BCPs in the fully optimized structures of Br_4–Br_{12} 13
Figure S5. Plot of θ_p versus θ for the non-covalent Br-∗-Br interactions at the BCPs in the fully optimized structures of Br_4–Br_{12} 13
Figure S6. Plot of $E(2)$ versus R for the non-covalent Br-∗-Br interactions in Br_4 (C_s-L_1)–Br_{12} (C_s-L_5) 14
Figure S7. Plot of $E(2)$ versus θ for the non-covalent Br-∗-Br interactions in Br_4 (C_s-L_1)–Br_{12} (C_s-L_5) 14
Figure S8. Plot of $E(2)$ versus θ_p for the non-covalent Br-∗-Br interactions in Br_4 (C_s-L_1)–Br_{12} (C_s-L_5) 15
Figure S9. MO_i (i = 70, 67, 64, 35 and 30) and the energies relative to those corresponding to $2Br_2$ 15
Computation information and geometries of compounds 16–19

Appendix

Survey of QTAIM, closely related to QTAIM dual functional analysis 20–22
Criteria for classification of interactions: behavior of typical interactions elucidated by QTAIM-DFA 23
Characterization of interactions 23–25
References 26

Table S1. Structural parameters for Br_2–Br_6, optimized with MP2/6-311+G(3df).[1]

Species (Sym)[d]	r_1 (Å)	r_2 (Å)	r_3 (Å)	θ_1 (°)	θ_2 (°)	θ_3 (°)	ϕ_1[2] (°)	ϕ_2[3] (°)	ϕ_3[4] (°)	ΔE_{ES}[5] (unit[8])	ΔE_{ZP}[6] (unit[8])	n_i[7]
Br_2 ($D_{\infty h}$)	2.2806									0.0	0.0	0
Br_4 (C_s-L_1)	2.2807	3.4055	2.2872	89.6	176.5		0.0			–10.7	–10.0	0
Br_4 (C_{2h})	2.2802	4.0638	3.8958	69.3	77.4	33.2	0.0			–8.0	–7.4	0
Br_4 (D_{2d})	2.2798	4.0512		73.7	32.7		85.1			–9.1	–8.4	0
Br_4 (C_s)TS	2.2798	3.9272	2.2813	75.2	130.4	55.3	0.0			–7.4	–6.9	1
Br_4 (C_1)TS	2.2801	4.0863	2.2801	71.1	99.8	71.1	-44.1	22.9	-44.1	–7.6	–7.1	1
Br_4 (C_1)TS	2.2812	3.8622	2.2800	98.9	33.8	75.5	31.1			–7.0	–6.5	1
Br_4 (C_{2v})TS	2.2836	3.7622	2.2823	162.3	35.3	72.3	180.0			–8.7	–8.2	1
Br_6 (C_s-L_2)[9]	2.2880	3.3891	2.2886	176.0	88.2	177.8	0.0	0.0		–22.6	–21.0	0
Br_6 (C_2)	2.2807	3.4298	2.2925	89.1	176.5		2.1	66.7	71.0	$–21.1_1$	$–19.6_8$	0
Br_6 (C_{3h}-c)	2.2857	3.5111		159.4	80.6		0.0	0.0		-29.1	–27.2	0
Br_6 (C_{2h})TS	2.2807	3.4298	2.2925	89.1	176.6		0.0	0.0	180.0	$–21.1_0$	$–19.6_8$	1[10]
Br_6 (C_{2v})TS	2.2807	3.4295	2.2925	89.1	176.5		0.0	0.0	0.0	$–21.1_0$	$–19.6_7$	1

[1] Structural parameters are defined in Figure 2. [2] $\phi_1 = \phi(^1Br^2Br^3Br^4Br)$. [3] $\phi_2 = \phi(^3Br^4Br^5Br^6Br)$. [4] $\phi_3 = \phi(^1Br^2Br^5Br^6Br)$. [5] ΔE (= $E(Br_{2k}) - k \times E(Br_2)$) on the energy surface. [6] ΔE (= $E(Br_{2k}) - k \times E(Br_2)$) corrected with the zero-point energy. [7] Number of imaginary frequency. [8] In kJ mol^{-1}. [9] (r_4, r_5, θ_4, ϕ_3) = (3.3627 Å, 2.2809 Å, 90.6°, 0.0°). [10] n_i = 0, if optimized with MP2/6-311+G(3d).

Table S2. Structural parameters for Br_8–Br_{12}, optimized with MP2/6-311+G(3df).[1]

Species	r_1	r_2	r_3	r_4	r_5	r_6	r_7	r_8	r_9	r_{10}	r_{11}
Br_8 (C_s-L_3)	2.2882	3.3849	2.2898	3.3418	2.2890	3.3593	2.2809				
Br_8 (S_4)	2.2906	3.3260									
Br_8 (S_4-Wm)	2.2906	3.3043	4.1061								
Br_{10} (C_s-L_4)	2.2883	3.3828	2.2900	3.3370	2.2901	3.3371	2.2891	3.3567	2.2809		
Br_{10} (C_2)	2.2809	3.3671	2.2884	3.4244	2.2934						
Br_{10} (C_2-c)	2.2880	3.5658	2.2863	3.4808	2.2873	3.4269	3.9507	3.9836			
Br_{12} (C_s-L_5)	2.2883	3.3818	2.2901	3.3346	2.2904	3.3321	2.2903	3.3339	2.2891	3.3558	2.2809
Br_{12} (C_i)	2.2907	3.3231	2.2907	3.3231	2.2907	3.3231					

[1] The r_n values are given in Å, those of θ and ϕ are in degrees and ΔE are in kJ mol^{-1}.

(Table S2 continued)

Species	θ_1	θ_2	θ_3	θ_4	θ_5	θ_6	θ_7	θ_8	θ_9	θ_{10}
Br_8 (C_s-L_3)	176.1	88.4	177.4	89.7	177.5	90.4				
Br_8 (S_4)	175.7	88.8								
Br_8 (S_4-Wm)	175.4	51.6	98.6							
Br_{10} (C_s-L_4)	176.1	88.4	177.5	89.8	177.2	89.6	177.5	90.4		
Br_{10} (C_2)	90.8	178.3	86.9	175.3						
Br_{10} (C_2-c)	84.4	152.0	80.5	160.7	80.5	158.9				
Br_{12} (C_s-L_5)	176.1	88.5	177.5	89.9	177.3	89.7	177.2	89.7	177.5	90.4
Br_{12} (C_i)	176.2	88.9	176.2	88.9						

[1] The r_n values are given in Å, those of θ and ϕ are in degrees and ΔE are in kJ mol^{-1}.

(Table S2 continued)

Species	ϕ_1[2]	ϕ_2[3]	ϕ_3[4]	ϕ_4	ϕ_5	ϕ_6	ϕ_7	ΔE_{ES}	ΔE_{ZP}
Br_8 (C_s-L_3)	0.0	0.0	0.0					-34.5	-32.1
Br_8 (S_4)	5.7[5]	-5.7[6]		132.2[7]				-48.2	-45.1
Br_8 (S_4-Wm)	-172.6[8]	-172.6[9]		128.0[10]	-51.0[11]	-52.1[12]		-52.0	-49.0
Br_{10} (C_s-L_4)	0.0	0.0	0.0					-46.5	-43.4
Br_{10} (C_2)	-2.4	3.1		63.0[13]				-44.6	-41.7
Br_{10} (C_2-c)	-14.3	-6.3[14]		12.1[15]	124.4[16]	25.3[17]	11.2[18]	-74.4	-70.4
Br_{12} (C_s-L_5)	0.0	0.0	0.0					-58.5	-54.6
Br_{12} (C_i)	10.3	-10.3		-95.2[19]	0.0[20]			-73.1	-68.4

[1] The r_n values are given in Å, those of θ and ϕ are in degrees and ΔE are in kJ mol^{-1}. [2] $\phi_1 = \phi(^{1}Br^{2}Br^{3}Br^{4}Br)$. [3] $\phi_2 = \phi(^{3}Br^{4}Br^{5}Br^{6}Br)$. [4] $\phi_2 = \phi(^{5}Br^{6}Br^{7}Br^{8}Br)$. [5] $\phi_1 = \phi(^{1}Br^{2}Br^{1'}Br^{2'}Br)$. [6] $\phi_2 = \phi(^{1'}Br^{2'}Br^{1''}Br^{2''}Br)$. [7] $\phi_4 = \phi(^{1}Br^{1'}Br^{1'''}Br^{1''}Br)$. [8] $\phi_1 = \phi(^{1'''}Br^{2'''}Br^{1}Br^{2}Br)$. [9] $\phi_1 = \phi(^{1}Br^{2}Br^{1'}Br^{2'}Br)$. [10] $\phi_4 = \phi(^{1'''}Br^{2'''}Br^{2}Br^{1}Br)$. [11] $\phi_5 = \phi(^{2'''}Br^{2}Br^{2'}Br^{2''}Br)$. [12] $\phi_6 = \phi(^{1'''}Br^{2'''}Br^{2}Br^{2'}Br)$. [13] $\phi_4 = \phi(^{3}Br^{4}Br^{7}Br^{8}Br)$. [14] $\phi_4 = \phi(^{3}Br^{4}Br^{5}Br^{1'}Br)$. [15] $\phi_4 = \phi(^{1}Br^{1'}Br^{5}Br^{4}Br)$. [16] $\phi_5 = \phi(^{4'}Br^{5'}Br^{5}Br^{1'}Br)$. [17] $\phi_6 = \phi(^{1'}Br^{1}Br^{2}Br^{3}Br)$. [18] $\phi_7 = \phi(^{2}Br^{3}Br^{4}Br^{5}Br)$. [19] $\phi_4 = \phi(^{1}Br^{2}Br^{5}Br^{6}Br)$. [20] $\phi_5 = \phi(^{1}Br^{2}Br^{1'}Br^{2'}Br)$.

Table S3. The bond path distances (r_{BP}) and the straight-line distances (R_{SL}) in the polybromide clusters, together with the differences between the two, evaluated with MP2/6-311+G(3df).

Species	BP	R_{SL} (^{A}Br, ^{B}Br)[1] (Å)	r_{BP-1} (^{A}Br, BCP) (Å)	r_{BP-2} (BCP, ^{B}Br) (Å)	r_{BP} (^{A}Br---^{B}Br)[2] (Å)	Δr_{BP}[3] (Å)
Br_2 ($D_{\infty h}$)	r_1	2.2806	1.1403	1.1403	2.2806	0.0000
Br_3^- ($D_{\infty h}$)	r_1	2.5594	1.3246	1.2348	2.5594	0.0000
Br_4 (C_s-L_1)	r_1	2.2807	1.1371	1.1436	2.2807	0.0000
	r_2	3.4055	1.7850	1.6211	3.4061	0.0006
	r_3	2.2872	1.1401	1.1471	2.2872	0.0000
Br_4 (C_{2h})	r_1	2.2802	1.1409	1.1393	2.2802	0.0000
	r_2	4.0638	2.0245	2.0455	4.0700	0.0062
	r_3	3.8958	1.9499	1.9499	3.8999	0.0041
Br_4 (D_{2d})	r_1	2.2798	1.1399	1.1399	2.2798	0.0000
	r_2	4.0512	2.0274	2.0280	4.0554	0.0042
Br_4 (C_s)$_{TS}$	r_1	2.2798	1.1384	1.1415	2.2798	0.0000
	r_2	3.9272	1.9562	1.9728	3.9290	0.0018
	r_3	2.2813	1.1396	1.1417	2.2813	0.0000
Br_4 (C_1)$_{TS}$	r_1	2.2801	1.1396	1.1405	2.2801	0.0000
	r_2	4.0863	2.0362	2.0526	4.0889	0.0025
	r_3	2.2801	1.1397	1.1405	2.2801	0.0000
Br_4 (C_1)$_{TS}$	r_1	2.2812	1.1415	1.1398	2.2813	0.0001
	r_2	3.8622	1.9211	1.9503	3.8713	0.0091
	r_3	2.2800	1.1414	1.1386	2.2800	0.0000
Br_4 (C_{2v})$_{TS}$	r_1	2.2836	1.1436	1.1400	2.2836	0.0000
	r_2	3.7622	1.8210	1.9550	3.7760	0.0138
	r_3	2.2823	1.1412	1.1412	2.2823	0.0000
Br_6 (C_s-L_2)	r_1	2.2880	1.1482	1.1398	2.2880	0.0000
	r_2	3.3891	1.6130	1.7768	3.3898	0.0007
	r_3	2.2886	1.1517	1.1370	2.2887	0.0001
	r_4	3.3627	1.5985	1.7646	3.3631	0.0004
	r_5	2.2809	1.1440	1.1369	2.2809	0.0000
Br_6 (C_2)	r_1	2.2807	1.1375	1.1433	2.2808	0.0001
	r_2	3.4298	1.7955	1.6349	3.4304	0.0006
	r_3	2.2925	1.1462	1.1462	2.2925	0.0000
Br_6 (C_{3h}-c)	r_1	2.2857	1.1491	1.1366	2.2857	0.0000
	r_2	3.5111	1.6955	1.8244	3.5199	0.0088
Br_6 (C_{2h})$_{TS}$	r_1	2.2807	1.1374	1.1433	2.2807	0.0000
	r_2	3.4298	1.7955	1.6348	3.4304	0.0006
	r_3	2.2925	1.1462	1.1462	2.2925	0.0000
Br_6 (C_{2v})$_{TS}$	r_1	2.2807	1.1374	1.1433	2.2807	0.0000
	r_2	3.4295	1.7954	1.6348	3.4301	0.0006
	r_3	2.2925	1.1462	1.1462	2.2925	0.0000
Br_8 (C_s-L_3)	r_1	2.2882	1.1485	1.1397	2.2882	0.0000
	r_2	3.3849	1.6109	1.7747	3.3857	0.0008
	r_3	2.2898	1.1531	1.1367	2.2898	0.0000
	r_4	3.3418	1.5881	1.7541	3.3422	0.0004
	r_5	2.2890	1.1522	1.1368	2.2890	0.0000
	r_6	3.3593	1.5968	1.7629	3.3597	0.0004
	r_7	2.2809	1.1442	1.1367	2.2809	0.0000

[1] R_{SL}(^{A}Br, ^{B}Br) is straight-line distance of the bonds or interactions between ^{A}Br and ^{B}Br (A < B) in Br_n. [2] r_{BP}(^{A}Br---^{B}Br) is bond path distance of the bonds or interactions between ^{A}Br and ^{B}Br (A < B) in Br_n; $r_{BP} = r_{BP-1} + r_{BP-2}$. [3] $\Delta r_{BP} = r_{BP}$(^{A}Br---^{B}Br)– R_{SL}(^{A}Br, ^{B}Br).

(Table S3 continued)

Species	BP	R_{SL} (^{A}Br, ^{B}Br)[1] (Å)	r_{BP-1} (^{A}Br, BCP) (Å)	r_{BP-2} (BCP, ^{B}Br) (Å)	r_{BP} (^{A}Br---^{B}Br)[2] (Å)	Δr_{BP}[3] (Å)
Br_8 (S_4)	r_1	2.2906	1.1551	1.1355	2.2906	0.0000
	r_2	3.3260	1.5793	1.7475	3.3268	0.0008
Br_8 (S_4-Wm)	r_1	2.2906	1.1468	1.1438	2.2906	0.0000
	r_2	3.3043	1.5718	1.7331	3.3050	0.0007
	r_3	4.1061	2.0600	2.0601	4.1201	0.0140
Br_{10} (C_s-L_4)	r_1	2.2883	1.1486	1.1397	2.2883	0.0000
	r_2	3.3828	1.6098	1.7737	3.3835	0.0007
	r_3	2.2900	1.1534	1.1366	2.2900	0.0000
	r_4	3.3370	1.5857	1.7517	3.3374	0.0004
	r_5	2.2901	1.1537	1.1365	2.2902	0.0001
	r_6	3.3371	1.5857	1.7518	3.3376	0.0005
	r_7	2.2891	1.1525	1.1366	2.2891	0.0000
	r_8	3.3567	1.5954	1.7617	3.3571	0.0004
	r_9	2.2809	1.1442	1.1367	2.2809	0.0000
Br_{10} (C_2-c)	r_1	2.2880	1.1440	1.1440	2.2880	0.0000
	r_2	3.5660	1.7368	1.8410	3.5779	0.0118
	r_3	2.2863	1.1499	1.1366	2.2865	0.0002
	r_4	3.4808	1.6778	1.8112	3.4890	0.0082
	r_5	2.2873	1.1508	1.1365	2.2873	0.0000
	r_6	3.4269	1.6550	1.7805	3.4355	0.0086
	r_7	3.9507	1.9909	1.9679	3.9588	0.0081
	r_8	3.9836	1.9919	1.9922	3.9841	0.0005
Br_{10} (C_2)	r_1	2.2809	1.1371	1.1439	2.2809	0.0000
	r_2	3.3671	1.7666	1.6008	3.3674	0.0003
	r_3	2.2884	1.1374	1.1510	2.2884	0.0000
	r_4	3.4244	1.7924	1.6330	3.4254	0.0010
	r_5	2.2934	1.1467	1.1467	2.2934	0.0000
Br_{12} (C_s-L_5)	r_1	2.2883	1.1487	1.1396	2.2883	0.0000
	r_2	3.3818	1.6093	1.7733	3.3826	0.0008
	r_3	2.2901	1.1536	1.1365	2.2901	0.0000
	r_4	3.3346	1.5845	1.7505	3.3350	0.0004
	r_5	2.2904	1.1540	1.1364	2.2904	0.0000
	r_6	3.3321	1.5832	1.7493	3.3325	0.0004
	r_7	2.2903	1.1540	1.1363	2.2903	0.0000
	r_8	3.3339	1.5841	1.7503	3.3344	0.0005
	r_9	2.2891	1.1526	1.1365	2.2891	0.0000
	r_{10}	3.3558	1.5949	1.7613	3.3562	0.0004
	r_{11}	2.2809	1.1443	1.1366	2.2809	0.0000
Br_{12} (C_i)	r_1	2.2907	1.1550	1.1357	2.2907	0.0000
	r_2	3.3231	1.5790	1.7447	3.3238	0.0007
	r_3	2.2907	1.1550	1.1357	2.2907	0.0000
	r_4	3.3231	1.5790	1.7447	3.3237	0.0006
	r_5	2.2907	1.1550	1.1358	2.2908	0.0001
	r_6	3.3231	1.5768	1.7474	3.3242	0.0011

[1] R_{SL}(^{A}Br, ^{B}Br) is straight-line distance of the bonds or interactions between ^{A}Br and ^{B}Br (A < B) in Br_n. [2] r_{BP}(^{A}Br---^{B}Br) is bond path distance of the bonds or interactions between ^{A}Br and ^{B}Br (A < B) in Br_n; $r_{BP} = r_{BP-1} + r_{BP-2}$. [3] $\Delta r_{BP} = r_{BP}$(^{A}Br---^{B}Br)– R_{SL}(^{A}Br, ^{B}Br).

Table S4. The $\rho_b(\boldsymbol{r}_c)$, $H_b(\boldsymbol{r}_c) - V_b(\boldsymbol{r}_c)/2$ (= $(\hbar^2/8m)\nabla^2\rho_b(\boldsymbol{r}_c)$), and $H_b(\boldsymbol{r}_c)$ values and QTAIM-DFA parameters for Br-*-Br in polybromine clusters of Br_2–Br_{12}, evaluated with MP2/6-311+G(3df).[1]

Species (Symmetry)[2]	BP on	$\rho_b(\boldsymbol{r}_c)$ (ea_o^{-3})	$c\nabla^2\rho_b(\boldsymbol{r}_c)$[3] (au)	$H_b(\boldsymbol{r}_c)$ (au)	$k_b(\boldsymbol{r}_c)$[4]	R (au)	θ (°)	C_{ii} (Å mDyn^{-1})	θ_p (°)	κ_p (au^{-1})
Br_2 ($D_{\infty h}$)	r_1	0.1154	-0.0044	-0.0574	-2.179	0.0576	184.3	0.366	190.9	0.3
Br_4 (C_s-L_1)	r_1	0.1154	-0.0043	-0.0574	-2.174	0.0576	184.2	0.368	191.0	0.3
Br_4 (C_s-L_1)	r_3	0.1140	-0.0038	-0.0558	-2.156	0.0559	183.9	0.383	191.0	0.3
Br_4 (C_{2h})	r_1	0.1155	-0.0044	-0.0575	-2.178	0.0576	184.3	0.367	190.9	0.3
Br_4 (D_{2d})	r_1	0.1156	-0.0044	-0.0576	-2.180	0.0578	184.4	0.366	190.9	0.3
Br_6 (C_s-L_2)	r_1	0.1138	-0.0037	-0.0556	-2.155	0.0558	183.8	0.385	191.0	0.3
Br_6 (C_s-L_2)	r_3	0.1137	-0.0036	-0.0555	-2.151	0.0556	183.7	0.389	191.0	0.3
Br_6 (C_s-L_2)	r_5	0.1154	-0.0043	-0.0574	-2.177	0.0575	184.3	0.368	190.9	0.3
Br_6 (C_2)	r_1	0.1154	-0.0045	-0.0574	-2.184	0.0576	184.4	0.367	191.4	0.4
Br_6 (C_2)	r_3	0.1129	-0.0033	-0.0546	-2.140	0.0547	183.5	0.395	191.0	0.3
Br_6 (C_{3h}-c)	r_1	0.1143	-0.0039	-0.0562	-2.160	0.0563	183.9	0.382	191.0	0.2
Br_8 (C_s-L_3)	r_1	0.1137	-0.0036	-0.0556	-2.150	0.0557	183.7	0.386	191.0	0.3
Br_8 (C_s-L_3)	r_3	0.1134	-0.0035	-0.0553	-2.147	0.0554	183.7	0.392	191.1	0.2
Br_8 (C_s-L_3)	r_5	0.1136	-0.0035	-0.0555	-2.146	0.0556	183.6	0.390	191.0	0.3
Br_8 (C_s-L_3)	r_7	0.1154	-0.0043	-0.0574	-2.177	0.0575	184.3	0.368	190.9	0.3
Br_8 (S_4)	r_1	0.1132	-0.0036	-0.0551	-2.151	0.0552	183.7	0.396	191.2	0.2
Br_8 (S_4-Wm[5])	r_1	0.1133	-0.0036	-0.0552	-2.148	0.0553	183.7	0.393	191.1	0.2
Br_{10} (C_s-L_4)	r_1	0.1137	-0.0037	-0.0556	-2.152	0.0557	183.8	0.386	191.0	0.3
Br_{10} (C_s-L_4)	r_3	0.1134	-0.0035	-0.0552	-2.147	0.0554	183.7	0.393	191.1	0.2
Br_{10} (C_s-L_4)	r_5	0.1133	-0.0035	-0.0552	-2.145	0.0553	183.6	0.394	191.1	0.2
Br_{10} (C_s-L_4)	r_7	0.1136	-0.0036	-0.0554	-2.149	0.0556	183.7	0.390	191.1	0.2
Br_{10} (C_s-L_4)	r_9	0.1154	-0.0043	-0.0574	-2.176	0.0575	184.3	0.368	191.0	0.3
Br_{10} (C_2-c)	r_1	0.1138	-0.0037	-0.0558	-2.153	0.0559	183.8	0.390	191.1	0.2
Br_{10} (C_2-c)	r_3	0.1141	-0.0036	-0.0561	-2.149	0.0562	183.7	0.384	191.0	0.3
Br_{10} (C_2-c)	r_5	0.1138	-0.0038	-0.0558	-2.157	0.0560	183.9	0.387	191.1	0.2
Br_{10} (C_2)	r_1	0.1154	-0.0042	-0.0574	-2.174	0.0575	184.2	0.368	190.9	0.3
Br_{10} (C_2)	r_3	0.1137	-0.0039	-0.0556	-2.164	0.0557	184.0	0.388	191.8	0.5
Br_{10} (C_2)	r_5	0.1127	-0.0033	-0.0544	-2.138	0.0545	183.5	0.398	191.0	0.4
Br_{12} (C_s-L_5)	r_1	0.1137	-0.0037	-0.0556	-2.154	0.0557	183.8	0.368	191.0	0.3
Br_{12} (C_s-L_5)	r_3	0.1133	-0.0035	-0.0552	-2.143	0.0553	183.6	0.394	191.1	0.3
Br_{12} (C_s-L_5)	r_5	0.1133	-0.0035	-0.0552	-2.146	0.0553	183.6	0.394	191.1	0.2
Br_{12} (C_s-L_5)	r_7	0.1133	-0.0034	-0.0552	-2.142	0.0553	183.6	0.394	191.1	0.2
Br_{12} (C_s-L_5)	r_9	0.1136	-0.0036	-0.0554	-2.149	0.0555	183.7	0.391	191.0	0.2
Br_{12} (C_s-L_5)	r_{11}	0.1154	-0.0042	-0.0574	-2.174	0.0575	184.2	0.368	191.0	0.3
Br_{12} (C_i)	r_1	0.1132	-0.0039	-0.0551	-2.165	0.0553	184.0	0.397	191.7	0.3
Br_{12} (C_i)	r_3	0.1132	-0.0035	-0.0551	-2.144	0.0553	183.6	0.397	191.1	0.2
Br_{12} (C_i)	r_5	0.1132	-0.0032	-0.0551	-2.132	0.0552	183.3	0.397	191.0	0.4

[1] The interactions in minima were tabulated. [2] Data are given at BCP. [3] $c\nabla^2\rho_b(\boldsymbol{r}_c) = H_b(\boldsymbol{r}_c) - V_b(\boldsymbol{r}_c)/2$, where $c = \hbar^2/8m$. [4] $k_b(\boldsymbol{r}_c) = V_b(\boldsymbol{r}_c)/G_b(\boldsymbol{r}_c)$. [5] Wind mill figure.

Table S5. Contributions from the donor-acceptor (NBO(*i*)→NBO(*j*)) interactions of the n(Br)→σ*(Br–Br) type in the optimized structures of Br_4–Br_{12} calculated with the NBO analysis under MP2/6-311+G(3df).

Species (symm) NBO(*j*)←NBO(*i*)	$E_s(2)$[1] (unit[5])	ΔE[2] (au)	$F(i,j)$[3]	NBO(*i*)	$E_p(2)$[1] (unit[5])	ΔE[2] (au)	$F(i,j)$[3]	$E_{s+p}(2)$[4] (unit[5])	$E_{s+p}(2)$[4] (unit[6])
Br_4 (C_s-L_1)									
σ*(^{3}Br–^{4}Br)←n_s(^{2}Br)	0.14	1.19	0.011	n_p(^{2}Br)	3.83	0.59	0.043	3.97	16.61
Br_6 (C_s-L_2)									
σ*(^{3}Br–^{4}Br)←n_s(^{2}Br)	0.14	1.19	0.012	n_p(^{2}Br)	4.08	0.59	0.044	4.22	17.66
σ*(^{5}Br–^{6}Br)←n_s(^{4}Br)	0.16	1.18	0.012	n_p(^{4}Br)	4.50	0.59	0.046	4.66	19.50
Br_8 (C_s-L_3)									
σ*(^{3}Br–^{4}Br)←n_s(^{2}Br)	0.14	1.19	0.012	n_p(^{2}Br)	4.15	0.59	0.044	4.29	17.95
σ*(^{5}Br–^{6}Br)←n_s(^{4}Br)	0.18	1.18	0.013	n_p(^{4}Br)	4.88	0.58	0.048	5.06	21.17
σ*(^{7}Br–^{8}Br)←n_s(^{6}Br)	0.17	1.18	0.013	n_p(^{6}Br)	4.56	0.59	0.046	4.73	19.79
Br_{10} (C_s-L_4)									
σ*(^{3}Br–^{4}Br)←n_s(^{2}Br)	0.15	1.19	0.012	n_p(^{2}Br)	4.19	0.59	0.044	4.34	18.16
σ*(^{5}Br–^{6}Br)←n_s(^{4}Br)	0.18	1.18	0.013	n_p(^{4}Br)	4.98	0.58	0.048	5.16	21.59
σ*(^{7}Br–^{8}Br)←n_s(^{6}Br)	0.18	1.18	0.013	n_p(^{6}Br)	4.97	0.58	0.048	5.15	21.55
σ*(^{9}Br–^{10}Br)←n_s(^{8}Br)	0.17	1.18	0.013	n_p(^{8}Br)	4.61	0.59	0.046	4.78	20.00
Br_{12} (C_s-L_5)									
σ*(^{3}Br–^{4}Br)←n_s(^{2}Br)	0.15	1.19	0.012	n_p(^{2}Br)	4.20	0.59	0.044	4.35	18.20
σ*(^{5}Br–^{6}Br)←n_s(^{4}Br)	0.18	1.18	0.013	n_p(^{4}Br)	5.02	0.58	0.048	5.20	21.76
σ*(^{7}Br–^{8}Br)←n_s(^{6}Br)	0.19	1.18	0.013	n_p(^{6}Br)	5.07	0.58	0.049	5.26	22.01
σ*(^{9}Br–^{10}Br)←n_s(^{8}Br)	0.18	1.18	0.013	n_p(^{8}Br)	5.04	0.58	0.048	5.22	21.84
σ*(^{11}Br–^{12}Br)←n_s(^{10}Br)	0.17	1.18	0.013	n_p(^{10}Br)	4.62	0.59	0.046	4.79	20.04

[1] Second order perturbation energy given by Equation (4) in the text. [2] The diagonal elements (orbital energies). [3] The off-diagonal NBO Fock matrix element. [4] $E_{s+p}(2) = E_s(2) + E_p(2)$. [5] kcal mol^{-1}. [6] kJ mol^{-1}.

Table S6. MO energies of Br_4 (C_{2h}), evaluated with MP2/6-311+G(3df).[1–3]

-490.08041	-490.08041	-490.07985	-490.07985	-65.21121
-65.21121	-65.21065	-65.21065	-58.57215	-58.57215
-58.57160	-58.57160	-58.56979	-58.56979	-58.56978
-58.56978	-58.56921	-58.56921	-58.56920	-58.56920
-9.88265	-9.88265	-9.88207	-9.88207	-7.49721
-7.49721	-7.49667	-7.49667	-7.48714	-7.48714
-7.48713	-7.48713	-7.48653	-7.48653	-7.48649
-7.48649	-3.23881	-3.23880	-3.23634	-3.23634
-3.23321	-3.23321	-3.23319	-3.23319	-3.23228
-3.23228	-3.23222	-3.23222	-3.22001	-3.22001
-3.22001	-3.22001	-3.21933	-3.21933	-3.21932
-3.21932	-1.10562	-1.10225	-0.94776	-0.94673
-0.53540	-0.53531	-0.52075	-0.50821	-0.50319
-0.49158	-0.40866	-0.40589	-0.40436	-0.40135

[1] In au. [2] The MO energies are given in the sequence, stating from that of ψ_1 to that of ψ_{70} (HOMO).
[3] $\sum_i{}^n \varepsilon_i$ = -6253.61194 au.

Table S7. MO energies of Br_4 (D_{2d}), evaluated with MP2/6-311+G(3df).[1–3]

-490.07925	-490.07925	-490.07925	-490.07925	-65.21005
-65.21005	-65.21005	-65.21005	-58.57099	-58.57099
-58.57099	-58.57099	-58.56862	-58.56862	-58.56862
-58.56862	-58.56861	-58.56861	-58.56861	-58.56861
-9.88150	-9.88150	-9.88145	-9.88145	-7.49614
-7.49614	-7.49598	-7.49598	-7.48595	-7.48595
-7.48594	-7.48594	-7.48593	-7.48593	-7.48592
-7.48592	-3.23790	-3.23790	-3.23548	-3.23548
-3.23225	-3.23225	-3.23222	-3.23222	-3.23147
-3.23147	-3.23145	-3.23145	-3.21881	-3.21880
-3.21880	-3.21880	-3.21876	-3.21875	-3.21875
-3.21874	-1.10539	-1.10109	-0.94631	-0.94631
-0.53448	-0.53407	-0.52203	-0.50547	-0.50547
-0.48800	-0.40732	-0.40413	-0.40413	-0.40128

[1] In au. [2] The MO energies are given in the sequence, stating from that of ψ_1 to that of ψ_{70} (HOMO).
[3] $\sum_i{}^n \varepsilon_i$ = -6253.48986 au.

Table S8. MO energies of Br_2 ($D_{\infty h}$), evaluated with MP2/6-311+G(3df).[1–3]

-490.08177	-490.08177	-65.21258	-65.21258	-58.57352
-58.57352	-58.57114	-58.57114	-58.57114	-58.57114
-9.88408	-9.88403	-7.49871	-7.49855	-7.48852
-7.48852	-7.48850	-7.48850	-3.24047	-3.23805
-3.23480	-3.23480	-3.23404	-3.23404	-3.22138
-3.22138	-3.22134	-3.22134	-1.10521	-0.94861
-0.53638	-0.50711	-0.50711	-0.40658	-0.40658

[1] In au. [2] The MO energies are given in the sequence, stating from that of ψ_1 to that of ψ_{35} (HOMO).
[3] $\sum_i^n \varepsilon_i$ = -3126.91786 au.

Table S9. MO energies of Br_4 (C_s-L_1), evaluated with MP2/6-311+G(3df).[1–3]

-490.09014	-490.08757	-490.0782	-490.07502	-65.22097
-65.21835	-65.20892	-65.20586	-58.58188	-58.57956
-58.57953	-58.57931	-58.57689	-58.57689	-58.56989
-58.56742	-58.56742	-58.56677	-58.56445	-58.56445
-9.89241	-9.88978	-9.88031	-9.87733	-7.50687
-7.50444	-7.49705	-7.49685	-7.49494	-7.49415
-7.49415	-7.49176	-7.48458	-7.48457	-7.48191
-7.48191	-3.24797	-3.24460	-3.24287	-3.24265
-3.24014	-3.24013	-3.23602	-3.23194	-3.23073
-3.23072	-3.22988	-3.22988	-3.22756	-3.22756
-3.22692	-3.22692	-3.21734	-3.21734	-3.21488
-3.21488	-1.11316	-1.09874	-0.95691	-0.94393
-0.54562	-0.54174	-0.51474	-0.50616	-0.50105
-0.49936	-0.41412	-0.41389	-0.40155	-0.40148

[1] In au. [2] The MO energies are given in the sequence, stating from that of ψ_1 to that of ψ_{70} (HOMO).
[3] $\sum_i^n \varepsilon_i$ = -6253.97176 au.

Table S10. The $\Delta\varepsilon_i$ values for Br_4 (C_s-L_1), relative to $2Br_2$ ($D_{\infty h}$), evaluated with MP2/6-311+G(3df).[1–3]

-21.975435	-15.227900	9.373035	17.722125	-22.027945
-15.149135	9.609330	17.643360	-21.949180	-15.858020
-15.779255	-15.201645	-15.096625	-15.096625	3.281875
9.766860	9.766860	11.473435	17.564595	17.564595
-21.870415	-14.965350	9.766860	17.590850	-21.424080
-15.044115	3.938250	4.463350	-16.855710	-14.781565
-14.781565	-8.506620	10.291960	10.318215	17.302045
17.302045	-19.691250	-10.843315	-12.654910	-12.077300
-14.020170	-13.993915	-3.203110	7.508930	8.690405
8.716660	10.922080	10.922080	-16.225590	-16.225590
-14.545270	-14.545270	10.502000	10.502000	16.960730
16.960730	-20.872725	16.986985	-21.791650	12.287340
-24.259620	-14.072680	-20.032565	2.494225	15.910530
20.347625	-19.796270	-19.192405	13.206265	13.390050

[1] In kJ mol^{-1}. [2] The $\Delta\varepsilon_i$ values are given in the sequence, stating from that of ψ_1 to that of ψ_{70} (HOMO) for Br_4 (C_s-L_1). [3] $\Delta\sum_i{}^n\varepsilon_i$ = -357.2 kJ mol^{-1}.

Table S11. Energies for the Br_4 clusters and $2Br_2$, together with the differences between the two.[1,2]

Species (symmetry)	E (ΔE)[3]	$\sum_i{}^n\varepsilon_i$ ($\Delta\sum_i{}^n\varepsilon_i$)[3]	$\sum_i{}^n H_c(i)$ ($\Delta\sum_i{}^n H_c(i)$)[3]	$(\sum_{i\neq j}{}^n J_{ij} - \sum_{i\neq j,\parallel}{}^n K_{ij})/2$ $\Delta((\sum_{i\neq j}{}^n J_{ij} - \sum_{i\neq j,\parallel}{}^n K_{ij})/2)$[3]
Br_4 (C_{2h})	-10290.13146	-6253.61194	-14326.65098	4036.51952
	(-0.00305)	(0.22378)	(-0.22988)	(0.22073)
	(-8.0)[4]	(587.5)[4]	(-603.5)[4]	(595.5)[4]
Br_4 (D_{2d})	-10290.13188	-6253.48986	-14326.7739	4036.64202
	(-0.00347)	(0.34586)	(-0.35280)	(0.34933)
	(-9.1)[4]	(908.1)[4]	(-926.3)[4]	(917.2)[4]
Br_4 (C_s-L_1)	-10290.13250	-6253.97176	-14326.29324	4036.16074
	(-0.00409)	(-0.13604)	(0.12786)	(-0.13195)
	(-10.7)[4]	(-357.2)[4]	(335.7)[4]	(-346.4)[4]
$2Br_2$ ($D_{\infty h}$)	-10290.12841	-6253.83572	-14326.4211	4036.29269
	(as 0.0)[4]	(as 0.0)[4]	(as 0.0)[4]	(as 0.0)[4]

[1] Evaluated with MP2/6-311+G(3df). [2] In au. [3] Values from $2Br_2$ ($D_{\infty h}$) are given in the parenthesis. [4] In kJ mol^{-1}.

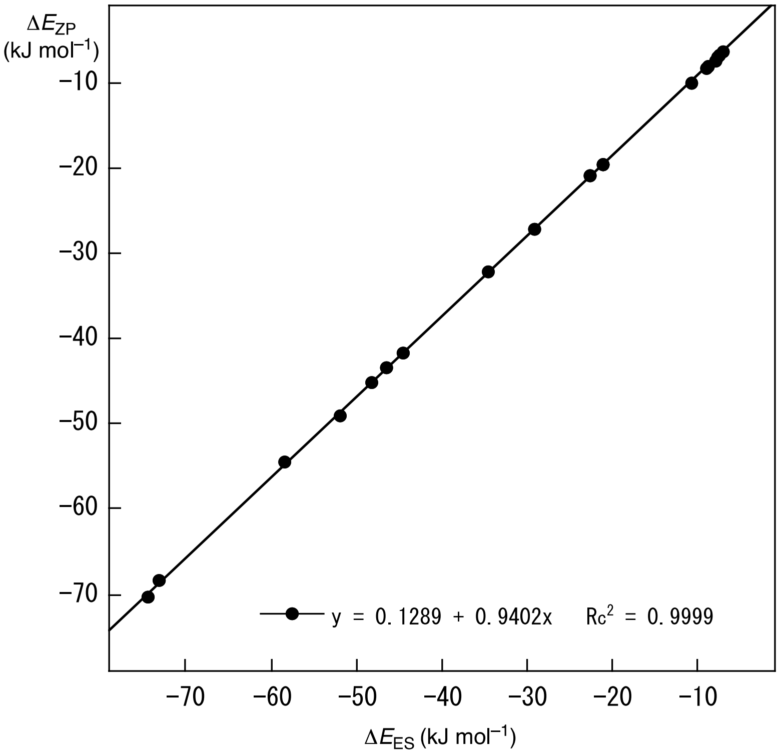

Figure S1. Plot of ΔE_{ZP} versus ΔE_{ES} for Br_4–Br_{12}, relative to those of Br_2, respectively.

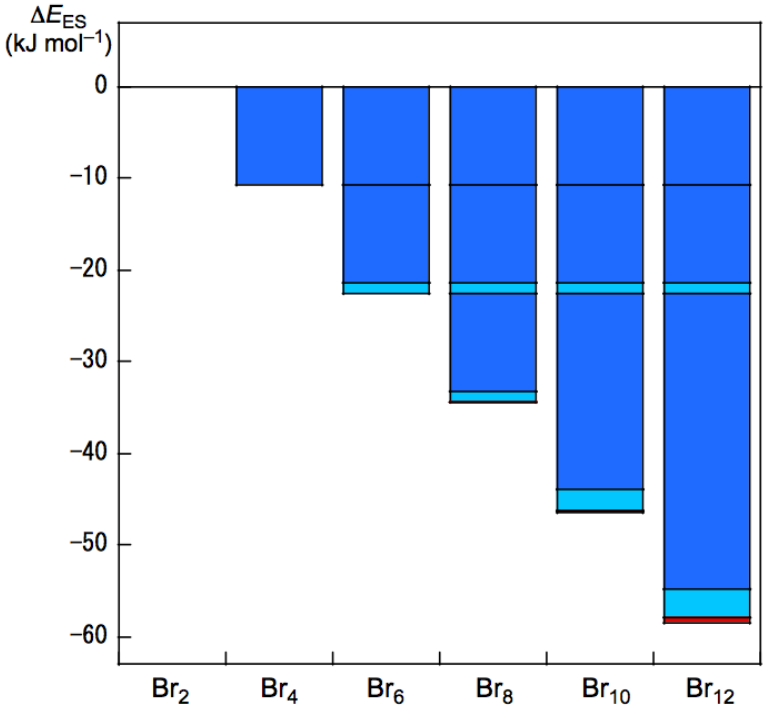

Figure S2. Plots of ΔE_{ES} for Br_2–Br_{12} (C_s-L_n). Contributions from ΔE_{ES} (Br_4) are shown by ■, from ΔE_{ES} (Br_6) by ■ + ■ and those inside contributions are by ■ + ■ + ■. The ■ area appears each column for Br_8–Br_{12}, although very slightly for Br_8 and Br_{10}, which shows to the contributions of ΔE_{ES} becomes larger in the order of ΔE_{ES} (Br_4) < (ΔE_{ES} (Br_6) – ΔE_{ES} (Br_4)) ≤ inside ΔE_{ES} for Br_8–Br_{12}.

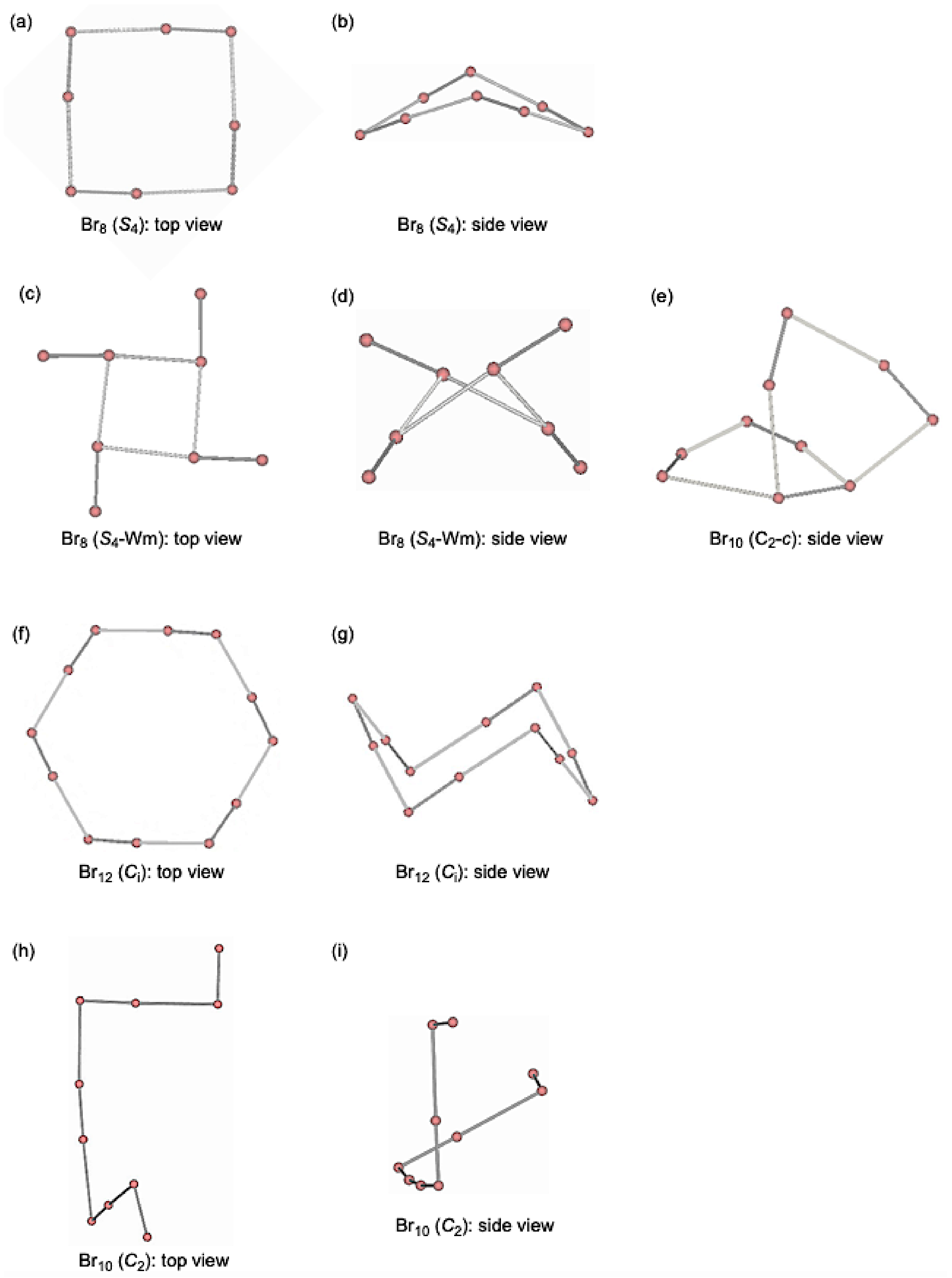

Figure S3. Optimized structures for the cyclic bromine clusters of Br_8–Br_{12}, together with the linear type bromine cluster of Br_{10}, calculated with MP2/6-311+G(3df).

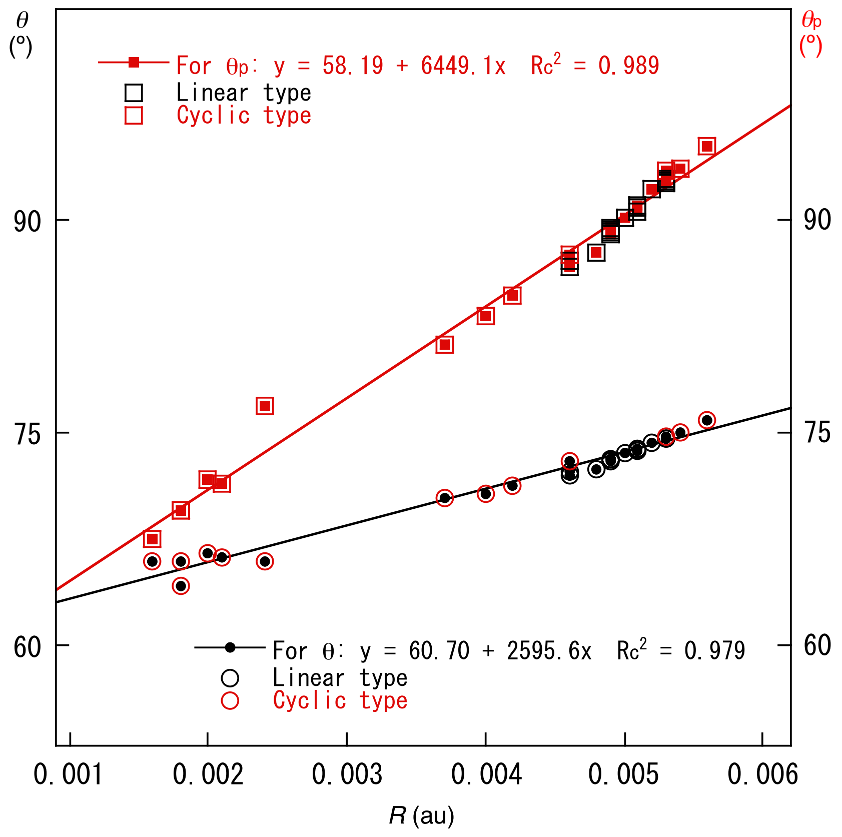

Figure S4. Plot of θ and θ_p versus R for the non-covalent Br-∗-Br interactions at the BCPs in the fully optimized structures of Br_4–Br_{12}. Correlations are given in the figure.

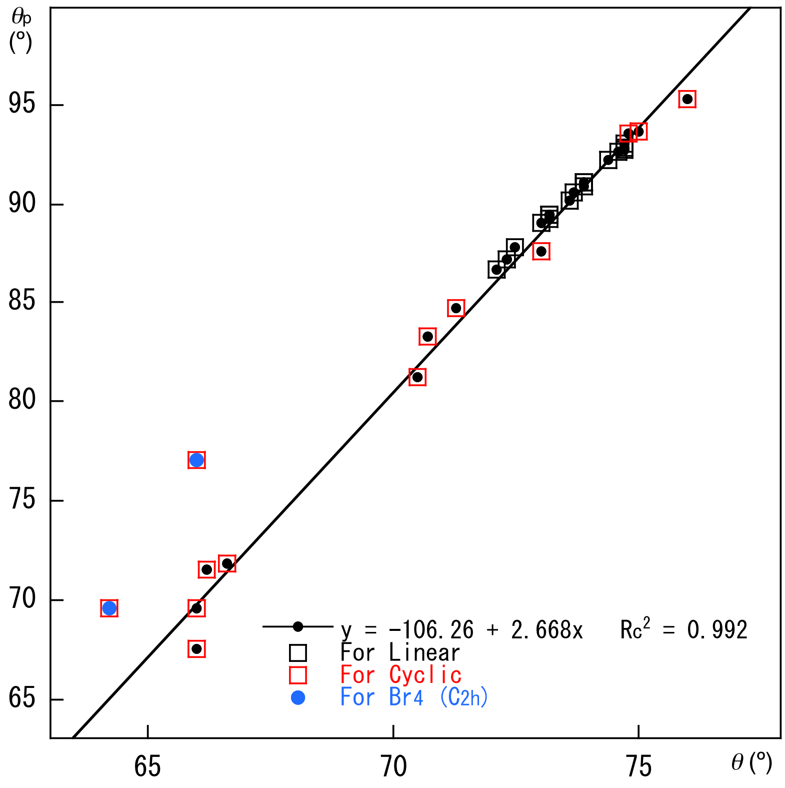

Figure S5. Plot of θ_p versus θ for the non-covalent Br-∗-Br interactions at the BCPs in the fully optimized structures of Br_4–Br_{12}. The correlation is given in the figure.

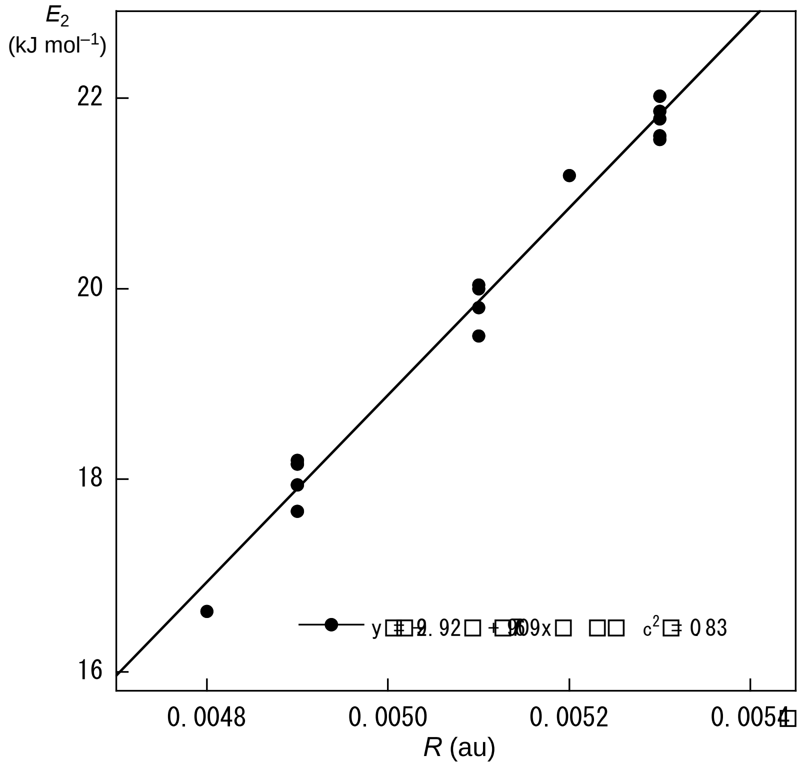

Figure S6. Plot of $E(2)$ versus R for the non-covalent Br-*-Br interactions in Br_4 (C_s-L_1)–Br_{12} (C_s-L_5), calculated with MP2/6-311+G(3df). The correlation is given in the figure.

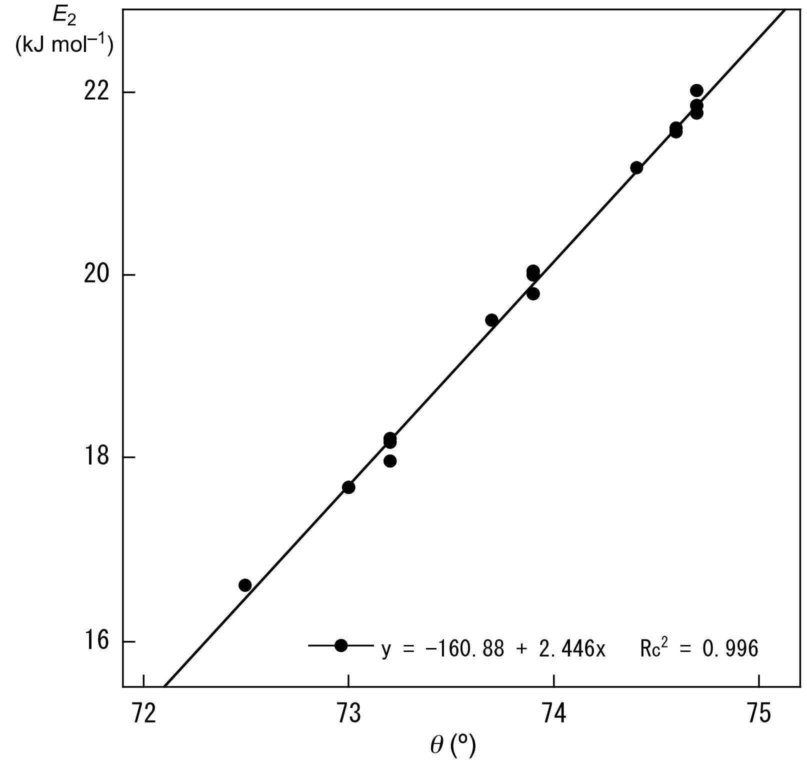

Figure S7. Plot of $E(2)$ versus θ for the non-covalent Br-*-Br interactions in Br_4 (C_s-L_1)–Br_{12} (C_s-L_5), calculated with MP2/6-311+G(3df). The correlation is given in the figure.

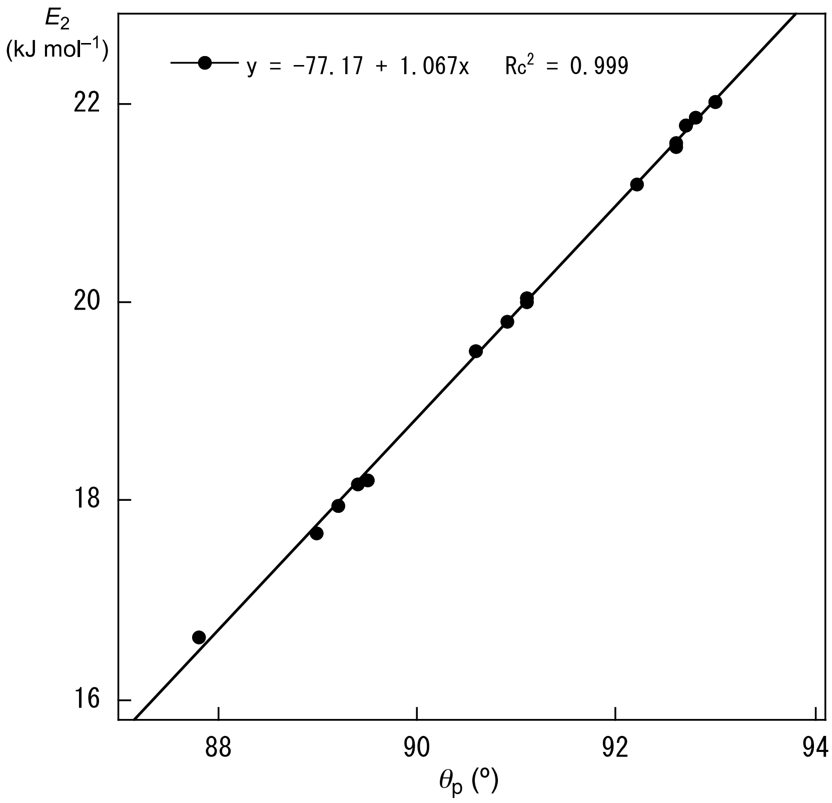

Figure S8. Plot of $E(2)$ versus θ_p for the non-covalent Br-∗-Br interactions in Br_4 (C_s-L_1)–Br_{12} (C_s-L_5), calculated with MP2/6-311+G(3df). The correlation is given in the figure.

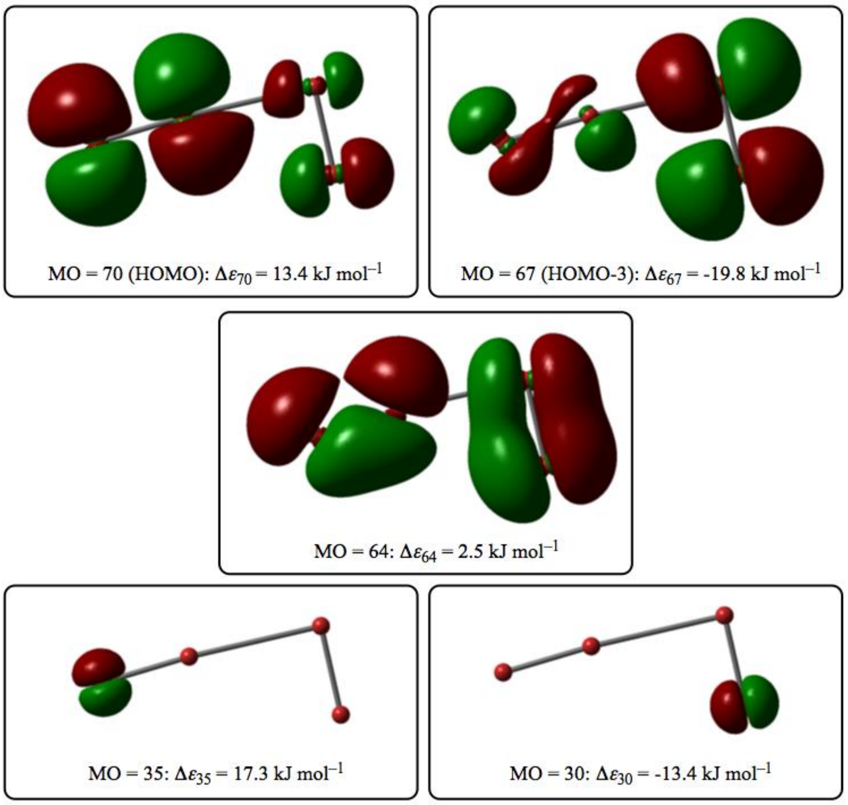

Figure S9. MO_i (i = 70, 67, 64, 35 and 30) and the energies relative to those corresponding to $2Br_2$.

Optimized structures given by Cartesian coordinates

Optimized structures given by Cartesian coordinates for examined molecules, together with the total energies with MP2/6-311+G(3df) method of the Gaussian 09 program. The optimized structures were confirmed by the frequency analysis.

MP2/6-311+G(3df)
Specie Br_4 (C_s-L_1)
Symmetry C_s
energy MP2 = -10290.1324977 au
Standard orientation

35	0	-0.407495	-2.784878	0.000000
35	0	1.609803	-1.720824	0.000000
35	0	0.000000	1.280008	0.000000
35	0	-1.202308	3.225694	0.000000

MP2/6-311+G(3df)
Specie Br_6 (C_s-L_2)
Symmetry C_s
energy MP2 =-15435.2012054 au
Standard orientation

35	0	3.382958	3.375048	0.000000
35	0	3.362728	1.094234	0.000000
35	0	0.000000	1.086946	0.000000
35	0	-2.287191	1.168322	0.000000
35	0	-2.303876	-2.220695	0.000000
35	0	-2.154620	-4.503857	0.000000

MP2/6-311+G(3df)
Specie Br_8 (C_s-L_3)
Symmetry C_s
energy MP2 = -20580.2699574 au
Standard orientation

35	0	-2.246779	-6.404496	0.000000
35	0	-3.373405	-4.421256	0.000000
35	0	-0.465337	-2.739637	0.000000
35	0	1.563545	-1.679916	0.000000
35	0	0.000000	1.273524	0.000000
35	0	-1.160973	3.247127	0.000000
35	0	1.803874	4.880304	0.000000
35	0	3.879074	5.844349	0.000000

MP2/6-311+G(3df)
Specie Br_{10} (C_s-L_4)
Symmetry C_s
energy MP2 = -25725.3387378 au
Standard orientation

35	0	-2.440025	-8.531026	0.000000
35	0	-0.614927	-7.150697	0.000000

35	0	1.938554	-4.931969	0.000000
35	0	0.390124	-3.244866	0.000000
35	0	-1.757302	-0.690650	0.000000
35	0	0.000000	0.777898	0.000000
35	0	2.454038	3.039276	0.000000
35	0	0.891195	4.711823	0.000000
35	0	-1.291565	7.261926	0.000000
35	0	0.429908	8.758286	0.000000

MP2/6-311+G(3df)
Specie Br_{12} (C_s-L_5)
Symmetry C_s
energy MP2 = -30870.4075278 au
Standard orientation

35	0	0.512403	10.862176	0.000000
35	0	1.829015	8.990545	0.000000
35	0	3.584135	6.099811	0.000000
35	0	1.595800	4.963565	0.000000
35	0	-1.225235	3.185559	0.000000
35	0	0.000000	1.250467	0.000000
35	0	1.648325	-1.645351	0.000000
35	0	-0.347100	-2.769491	0.000000
35	0	-3.169107	-4.544668	0.000000
35	0	-1.938565	-6.474919	0.000000
35	0	-0.261318	-9.381495	0.000000
35	0	-2.228353	-10.536198	0.000000

MP2/6-311+G(3df)
Specie Br_6 (C_2)
Symmetry C_2
energy MP2 = -15435.2006538 au
Standard orientation

35	0	0.664554	4.574552	1.292638
35	0	-0.664554	4.522621	-0.560022
35	0	-0.194747	1.129574	-0.732616
35	0	0.194747	-1.129574	-0.732616
35	0	0.664554	-4.522621	-0.560022
35	0	-0.664554	-4.574552	1.292638

MP2/6-311+G(3df)
Specie Br_{10} (C_2)
Symmetry C_2
energy MP2 = -25725.3380218 au
Standard orientation

35	0	0.290076	6.782823	2.862349
35	0	1.282635	4.767747	2.466179
35	0	-0.446337	4.425735	-0.402788
35	0	-1.652852	4.251584	-2.339486
35	0	-0.446337	1.056278	-2.586254
35	0	0.446337	-1.056278	-2.586254
35	0	1.652852	-4.251584	-2.339486

35	0	0.446337	-4.425735	-0.402788
35	0	-1.282635	-4.767747	2.466179
35	0	-0.290076	-6.782823	2.862349

MP2/6-311+G(3df)
Specie Br_4 (C_{2h})
Symmetry C_{2h}
energy MP2 = -10290.1314558 au
Standard orientation

35	0	1.901330	1.856723	0.000000
35	0	1.901330	-0.423437	0.000000
35	0	-1.901330	-1.856723	0.000000
35	0	-1.901330	0.423437	0.000000

MP2/6-311+G(3df)
Specie Br_4 (D_{2d})
Symmetry D_{2d}
energy MP2 = -10290.1318778 au
Standard orientation

35	0	0.000000	1.139888	1.858334
35	0	0.000000	-1.139888	1.858334
35	0	-1.139888	0.000000	-1.858334
35	0	1.139888	0.000000	-1.858334

MP2/6-311+G(3df)
Specie Br_6 (C_{3h}-c)
Symmetry C_{3h}
energy MP2 = -15435.2037071 au
Standard orientation

35	0	0.000000	2.230225	0.000000
35	0	-2.280892	2.378551	0.000000
35	0	-1.931432	-1.115113	0.000000
35	0	-0.919440	-3.164586	0.000000
35	0	1.931432	-1.115113	0.000000
35	0	3.200332	0.786035	0.000000

MP2/6-311+G(3df)
Specie Br_8 (S_4)
Symmetry S_4
energy MP2 = -20580.2751657 au
Standard orientation

35	0	2.553906	2.796826	0.838944
35	0	0.374259	2.796826	0.134571
35	0	-2.796826	2.553906	-0.838944
35	0	-2.796826	0.374259	-0.134571
35	0	-2.553906	-2.796826	0.838944
35	0	-0.374259	-2.796826	0.134571
35	0	2.796826	-2.553906	-0.838944
35	0	2.796826	-0.374259	-0.134571

MP2/6-311+G(3df)
Specie Br_8 (S_4-Wm)
Symmetry S_4
energy MP2 = -20580.2766322 au
Standard orientation

35	0	-2.031782	3.124066	1.806844
35	0	-0.294784	2.031782	0.788759
35	0	3.124066	2.031782	-1.806844
35	0	2.031782	0.294784	-0.788759
35	0	2.031782	-3.124066	1.806844
35	0	0.294784	-2.031782	0.788759
35	0	-3.124066	-2.031782	-1.806844
35	0	-2.031782	-0.294784	-0.788759

MP2/6-311+G(3df)
Specie Br_{10} (C_2-*c*)
Symmetry C_2
energy MP2 = -25725.3493438 au
Standard orientation

35	0	-0.379330	-1.079262	2.470997
35	0	-1.180905	-4.135223	0.817156
35	0	0.379330	-3.316537	-0.639766
35	0	2.543115	-1.105669	-2.235117
35	0	1.985400	0.159858	-0.413269
35	0	0.379330	1.079262	2.470997
35	0	1.180905	4.135223	0.817156
35	0	-0.379330	3.316537	-0.639766
35	0	-2.543115	1.105669	-2.235117
35	0	-1.985400	-0.159858	-0.413269

MP2/6-311+G(3df)
Specie Br_{12} (C_i)
Symmetry C_i
energy MP2 = -30870.41309 au
Standard orientation

35	0	2.096821	-3.941774	1.699011
35	0	3.104278	-2.461509	0.270358
35	0	4.463857	-0.152307	-1.694819
35	0	3.684198	1.458049	-0.264403
35	0	2.362164	3.786953	1.703136
35	0	0.579133	3.919149	0.271168
35	0	-2.096821	3.941774	-1.699011
35	0	-3.104278	2.461509	-0.270358
35	0	-4.463857	0.152307	1.694819
35	0	-3.684198	-1.458049	0.264403
35	0	-2.362164	-3.786953	-1.703136
35	0	-0.579133	-3.919149	-0.271168

Appendix

QTAIM Dual Functional Analysis (QTAIM-DFA)

The bond critical point (BCP; ∗) is an important concept in QTAIM. The BCP of (ω, σ) = (3, –1)[S1] is a point along the bond path (BP) at the interatomic surface, where charge density $\rho(\boldsymbol{r})$ reaches a minimum. It is donated by $\rho_b(\boldsymbol{r}_c)$, so are other QTAIM functions, such as the total electron energy densities $H_b(\boldsymbol{r}_c)$, potential energy densities $V_b(\boldsymbol{r}_c)$ and kinetic energy densities $G_b(\boldsymbol{r}_c)$ at the BCPs. A chemical bond or interaction between A and B is denoted by A–B, which corresponds to the BP between A and B in QTAIM. We will use A-∗-B for BP, where the asterisk emphasizes the presence of a BCP in A–B.

The sign of the Laplacian $\rho_b(\boldsymbol{r}_c)$ ($\nabla^2\rho_b(\boldsymbol{r}_c)$) indicates that $\rho_b(\boldsymbol{r}_c)$ is depleted or concentrated with respect to its surrounding, since $\nabla^2\rho_b(\boldsymbol{r}_c)$ is the second derivative of $\rho_b(\boldsymbol{r}_c)$. $\rho_b(\boldsymbol{r}_c)$ is locally depleted relative to the average distribution around $\boldsymbol{r}_c$ if $\nabla^2\rho_b(\boldsymbol{r}_c) > 0$, but it is concentrated when $\nabla^2\rho_b(\boldsymbol{r}_c) < 0$. Total electron energy densities at BCPs ($H_b(\boldsymbol{r}_c)$) must be a more appropriate measure for weak interactions on the energy basis.[S1–S8] $H_b(\boldsymbol{r}_c)$ are the sum of kinetic energy densities ($G_b(\boldsymbol{r}_c)$) and potential energy densities ($V_b(\boldsymbol{r}_c)$) at BCPs, as shown in eqn (S1). Electrons at BCPs are stabilized when $H_b(\boldsymbol{r}_c) < 0$, therefore, interactions exhibit the covalent nature in this region, whereas they exhibit no covalency if $H_b(\boldsymbol{r}_c) > 0$, due to the destabilization of electrons at BCPs under the conditions.[S1] Eqn (S2) represents the relation between $\nabla^2\rho_b(\boldsymbol{r}_c)$ and $H_b(\boldsymbol{r}_c)$, together with $G_b(\boldsymbol{r}_c)$ and $V_b(\boldsymbol{r}_c)$, which is closely related to the virial theorem.

$$H_b(\boldsymbol{r}_c) = G_b(\boldsymbol{r}_c) + V_b(\boldsymbol{r}_c) \qquad \text{(SA1)}$$

$$(\hbar^2/8m)\nabla^2\rho_b(\boldsymbol{r}_c) = H_b(\boldsymbol{r}_c) - V_b(\boldsymbol{r}_c)/2 \qquad \text{(SA2)}$$

$$= G_b(\boldsymbol{r}_c) + V_b(\boldsymbol{r}_c)/2 \qquad \text{(SA2')}$$

Interactions are classified by the signs of $\nabla^2\rho_b(\boldsymbol{r}_c)$ and $H_b(\boldsymbol{r}_c)$. Interactions in the region of $\nabla^2\rho_b(\boldsymbol{r}_c) < 0$ are called shared-shell (SS) interactions and they are closed-shell (CS) interactions for $\nabla^2\rho_b(\boldsymbol{r}_c) > 0$. $H_b(\boldsymbol{r}_c)$ must be negative when $\nabla^2\rho_b(\boldsymbol{r}_c) < 0$, since $H_b(\boldsymbol{r}_c)$ are larger than $(\hbar^2/8m)\nabla^2\rho_b(\boldsymbol{r}_c)$ by $V_b(\boldsymbol{r}_c)/2$ with negative $V_b(\boldsymbol{r}_c)$ at all BCPs (eqn (S2)). Consequently, $\nabla^2\rho_b(\boldsymbol{r}_c) < 0$ and $H_b(\boldsymbol{r}_c) < 0$ for the SS interactions. The CS interactions are especially called *pure* CS interactions for $H_b(\boldsymbol{r}_c) > 0$ and $\nabla^2\rho_b(\boldsymbol{r}_c) > 0$, since electrons at BCPs are depleted and destabilized under the conditions.[S1a] Electrons in the intermediate region between SS and *pure* CS, which belong to CS, are locally depleted but stabilized at BCPs, since $\nabla^2\rho_b(\boldsymbol{r}_c) > 0$ but $H_b(\boldsymbol{r}_c) < 0$.[S1a] We call the interactions in this region *regular* CS,[S4,S5] when it is necessary to distinguish from *pure* CS. The role of $\nabla^2\rho_b(\boldsymbol{r}_c)$ in the classification can be replaced by $H_b(\boldsymbol{r}_c) - V_b(\boldsymbol{r}_c)/2$, since $(\hbar^2/8m)\nabla^2\rho_b(\boldsymbol{r}_c) = H_b(\boldsymbol{r}_c) - V_b(\boldsymbol{r}_c)/2$ (eqn (S2)). Scheme SA1 summarizes the classification.

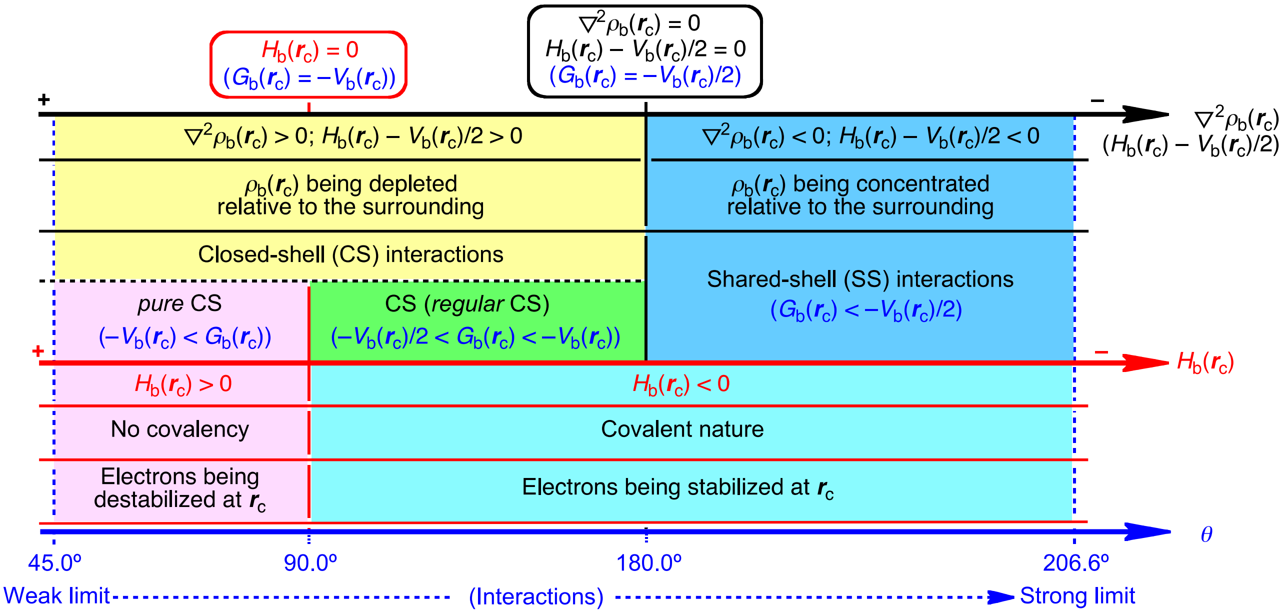

Scheme SA1. Classification of interactions by the signs of $\nabla^2\rho_b(\boldsymbol{r}_c)$ and $H_b(\boldsymbol{r}_c)$, together with $G_b(\boldsymbol{r}_c)$ and $V_b(\boldsymbol{r}_c)$.

We proposed QTAIM-DFA by plotting $H_b(\boldsymbol{r}_c)$ versus $H_b(\boldsymbol{r}_c) - V_b(\boldsymbol{r}_c)/2$ (= $(\hbar^2/8m)\nabla^2\rho_b(\boldsymbol{r}_c)$),[S4a] after the proposal of $H_b(\boldsymbol{r}_c)$ versus $\nabla^2\rho_b(\boldsymbol{r}_c)$.[S4b] Both axes in the plot of the former are given in energy unit, therefore, distances on the (x, y) (= $(H_b(\boldsymbol{r}_c) - V_b(\boldsymbol{r}_c)/2, H_b(\boldsymbol{r}_c))$) plane can be expressed in the energy unit, which provides an analytical development. QTAIM-DFA incorporates the classification of interactions by the signs of $\nabla^2\rho_b(\boldsymbol{r}_c)$ and $H_b(\boldsymbol{r}_c)$. Scheme SA2 summarizes the QTAIM-DFA treatment. Interactions of *pure* CS appear in the first quadrant, those of *regular* CS in the fourth quadrant and SS interactions do in the third quadrant. No interactions appear in the second one.

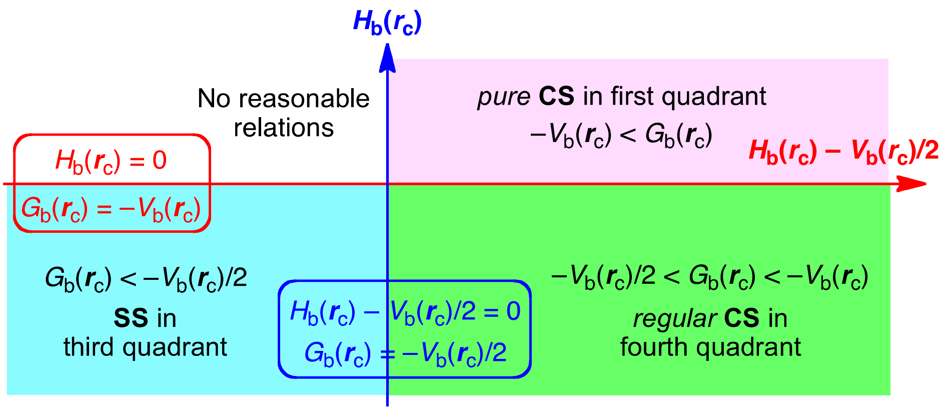

Scheme SA2. QTAIM-DFA: Plot of $H_b(\boldsymbol{r}_c)$ versus $H_b(\boldsymbol{r}_c) - V_b(\boldsymbol{r}_c)/2$ for Weak to Strong Interactions.

In our treatment, data for perturbed structures around fully optimized structures are also employed for the plots, together with the fully optimized ones (see Figure S1).[S4–S8] We proposed the concept of the "dynamic nature of interaction" originated from the perturbed structures. The behavior of interactions at the fully optimized structures corresponds to "the static nature of interactions", whereas that containing perturbed structures exhibit the "dynamic nature of interaction" as explained below. The method to generate the perturbed structures is discussed later. Plots of $H_b(\boldsymbol{r}_c)$ versus $H_b(\boldsymbol{r}_c) - V_b(\boldsymbol{r}_c)/2$ are analyzed employing

the polar coordinate (R, θ) representation with (θ_p, κ_p) parameters.[S4a,S5–S8] Figure S1 explains the treatment. R in (R, θ) is defined by eqn (S3) and given in the energy unit. Indeed, R does not correspond to the usual interaction energy, but it does to the local energy at BCP, expressed by $[(H_b(\boldsymbol{r}_c))^2 + (H_b(\boldsymbol{r}_c) - V_b(\boldsymbol{r}_c)/2)^2]^{1/2}$ in the plot (cf: eqn (S3)), where $R = 0$ for the enough large interaction distance. The plots show a spiral stream, as a whole. θ in (R, θ) defined by eqn (S4), measured from the y-axis, controls the spiral stream of the plot. Each plot for an interaction shows a specific curve, which provides important information of the interaction (see Figure S1). The curve is expressed by θ_p and κ_p. While θ_p, defined by eqn (S5) and measured from the y-direction, corresponds to the tangent line of a plot, where θ_p is calculated employing data of the perturbed structures with a fully-optimized structure and κ_p is the curvature of the plot (eqn (S6)). While (R, θ) correspond to the static nature, (θ_p, κ_p) represent the dynamic nature of interactions. We call (R, θ) and (θ_p, κ_p) QTAIM-DFA parameters, whereas $\rho_b(\boldsymbol{r}_c)$, $\nabla^2\rho_b(\boldsymbol{r}_c)$, $G_b(\boldsymbol{r}_c)$, $V_b(\boldsymbol{r}_c)$, $H_b(\boldsymbol{r}_c)$ and $H_b(\boldsymbol{r}_c) - V_b(\boldsymbol{r}_c)/2$ belong to QTAIM functions. $k_b(\boldsymbol{r}_c)$, defined by eqn (S7), is an QTAIM function but it will be treated as if it were an QTAIM-DFA parameter, if suitable.

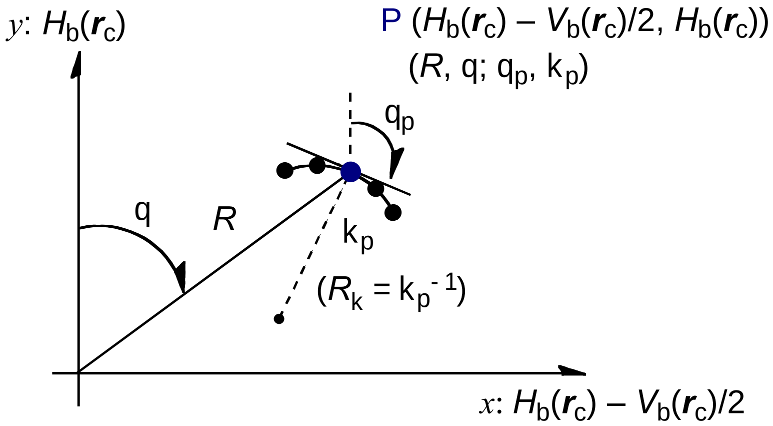

Figure SA1. Polar (R, θ) coordinate representation of $H_b(r_c)$ versus $H_b(r_c) - V_b(r_c)/2$, with (θ_p, κ_p) parameters.

$$R = (x^2 + y^2)^{1/2} \quad \text{(SA3)}$$
$$\theta = 90^\circ - \tan^{-1}(y/x) \quad \text{(SA4)}$$
$$\theta_p = 90^\circ - \tan^{-1}(dy/dx) \quad \text{(SA5)}$$
$$\kappa_p = |d^2y/dx^2| / [1 + (dy/dx)^2]^{3/2} \quad \text{(SA6)}$$
$$k_b(\boldsymbol{r}_c) = V_b(\boldsymbol{r}_c)/G_b(\boldsymbol{r}_c) \quad \text{(SA7)}$$

where $(x, y) = (H_b(\boldsymbol{r}_c) - V_b(\boldsymbol{r}_c)/2, H_b(\boldsymbol{r}_c))$

Criteria for Classification of Interactions: Behavior of Typical Interactions Elucidated by QTAIM-DFA

$H_b(\boldsymbol{r}_c)$ are plotted versus $H_b(\boldsymbol{r}_c) - V_b(\boldsymbol{r}_c)/2$ for typical interactions in vdW (van der Waals interactions), HBs (hydrogen bonds), CT-MCs (molecular complexes through charge transfer), X_3^- (trihalide ions), CT-TBPs (trigonal bipyramidal adducts through charge-transfer), Cov-w (weak covalent bonds) and Cov-s (strong covalent bonds).[S4–S8] Rough criteria are obtained by applying QTAIM-DFA, after the analysis of the plots for the typical interactions according to eqns (S3)–(S7). Scheme SA3 shows the rough criteria, which are accomplished by the θ and θ_p values, together with the values of $k_b(\boldsymbol{r}_c)$. The criteria will be employed to discuss the nature of interactions in question, as a reference.

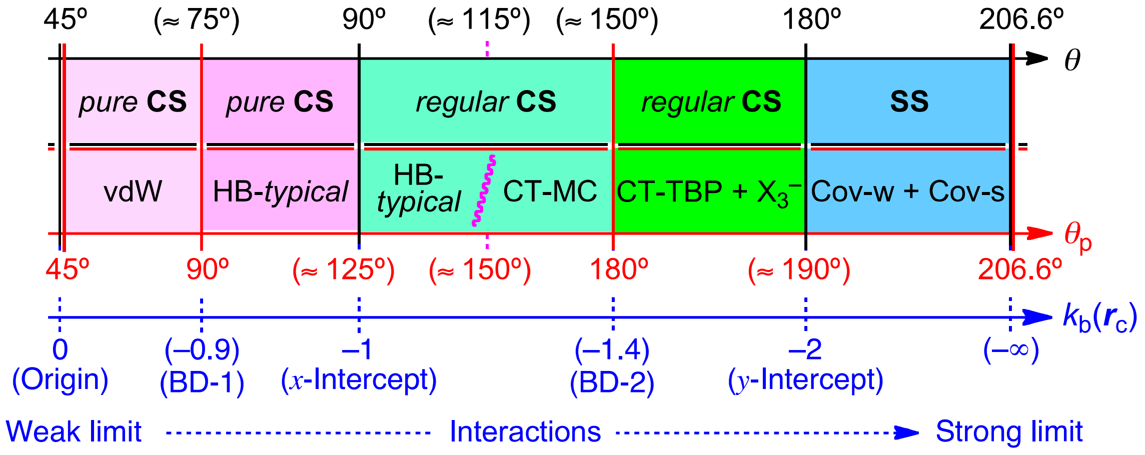

Scheme SA3. Rough classification and characterization of interactions by θ and θ_p, together with $k_b(\boldsymbol{r}_c)$ (= $V_b(\boldsymbol{r}_c)/G_b(\boldsymbol{r}_c)$).

Characterization of interactions

The characterization of interactions is explained employing $[^1Cl\text{-}^2Cl\text{-}^3Cl]^-$. The wide range of the perturbed structures were generated by partially optimizing $r(^2Cl\text{-}^3Cl)$ in $[^1Cl\text{-}^2Cl\text{-}^3Cl]^-$, assuming the $C_{\infty v}$ symmetry, with $r(^1Cl\text{-}^2Cl)$ being fixed in the wide range. The partial optimization method is called POM.[S4b,S5] The QTAIM functions, such as $V_b(\boldsymbol{r}_c)$, $G_b(\boldsymbol{r}_c)$, $H_b(\boldsymbol{r}_c)$, $H_b(\boldsymbol{r}_c) - V_b(\boldsymbol{r}_c)/2$ are calculated at BCPs for the wide varieties of the perturbed structures of $[^1Cl\text{-}^2Cl\text{-}^3Cl]^-$. $H_b(\boldsymbol{r}_c) - V_b(\boldsymbol{r}_c)/2$ and $H_b(\boldsymbol{r}_c)$ are plotted versus the interaction distances $r(^1Cl\text{-}^2Cl)$ in the perturbed structures of $[^1Cl\text{-}^2Cl\text{-}^3Cl]^-$, in the wide range. Figure S2 shows the plots. Each plot is analyzed using a regression curve of the ninth function and the first derivative of each regression curve is obtained. As shown in Figure S2, the maximum value of $H_b(\boldsymbol{r}_c)$ ($\mathrm{d}(H_b(\boldsymbol{r}_c)/\mathrm{d}r = 0$) is defined as the borderline between vdW and t-HB interactions. Similarly, the maximum value of $H_b(\boldsymbol{r}_c) - V_b(\boldsymbol{r}_c)/2$ ($\mathrm{d}(H_b(\boldsymbol{r}_c) - V_b(\boldsymbol{r}_c)/2)/\mathrm{d}r = 0$) does to the borderline between CT-MC and CT-TBP. However, it seems difficult to find a characteristic point corresponding to the borderline between *t*-HB and CT-MC in nature. Therefore, the borderline is tentatively given by $\theta_p = 150°$ based on the expectation form the experimental results, where θ_p is defined by $[90° - \tan^{-1}[\mathrm{d}H_b(\boldsymbol{r}_c)/\mathrm{d}(H_b(\boldsymbol{r}_c) - V_b(\boldsymbol{r}_c)/2)]]$ in the plot of $H_b(\boldsymbol{r}_c)$ versus $H_b(\boldsymbol{r}_c) - V_b(\boldsymbol{r}_c)/2$. The proposed classification and characterization of interactions, by means of the QTAIM functions of $H_b(\boldsymbol{r}_c)$, $H_b(\boldsymbol{r}_c) - V_b(\boldsymbol{r}_c)/2$, $G_b(\boldsymbol{r}_c)$ and/or $V_b(\boldsymbol{r}_c)$, are summarized in Table S1. The plot of $H_b(\boldsymbol{r}_c) - V_b(\boldsymbol{r}_c)/2$ versus w in Figure S2 is essentially the same as that of $\nabla^2\rho_b(\boldsymbol{r}_c)$ versus d(H---F) in X–H---F–Y, presented by Espinosa and co-workers.[S9]

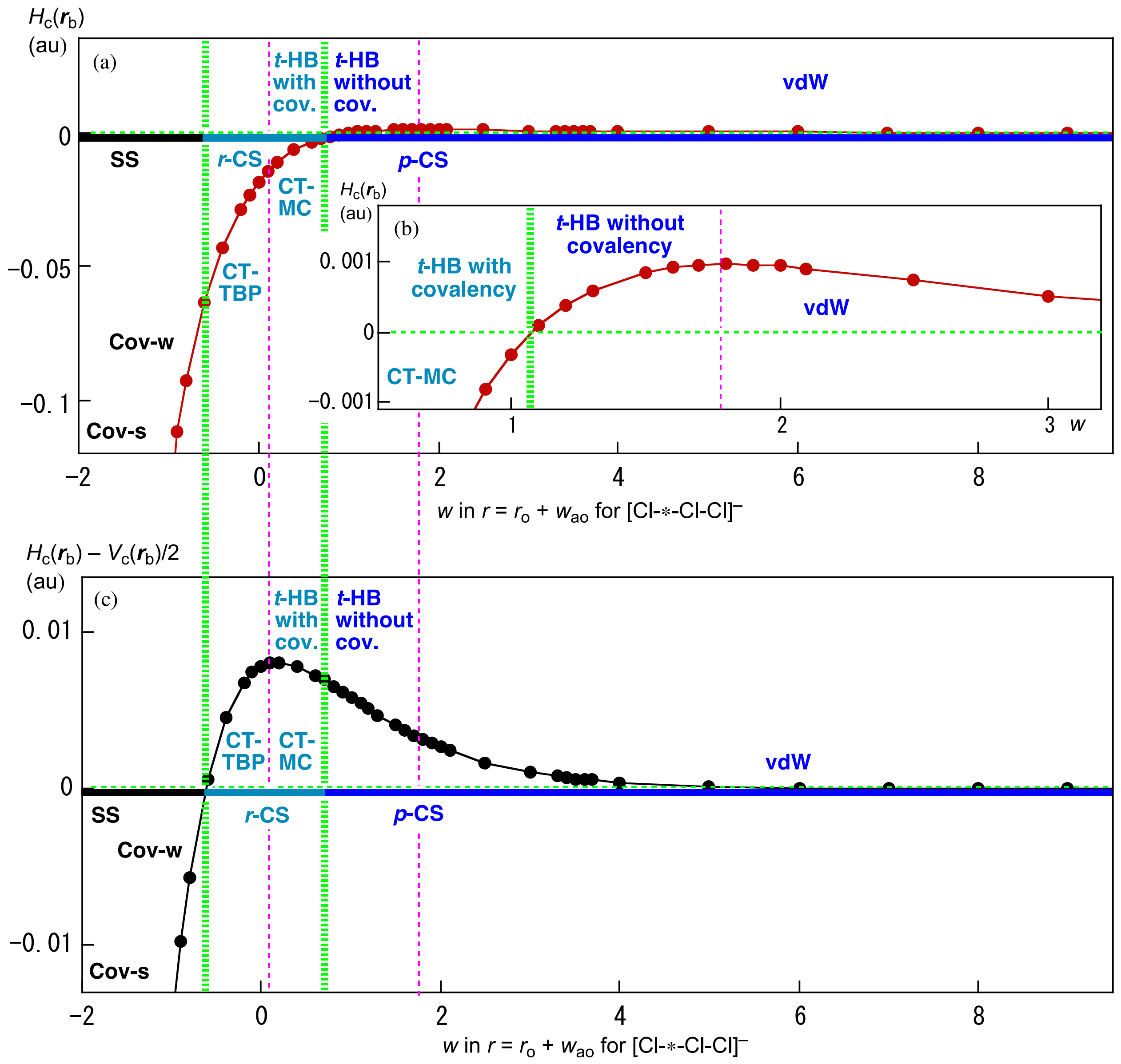

Figure SA2. Plot of $H_b(\boldsymbol{r}_c)$ versus w in $r(^1Cl\text{-}^2Cl) = r_o(^1Cl\text{-}^2Cl) + wa_o$ for $^1Cl\text{-}^2Cl\text{-}^3Cl^-$ (a) with the magnified picture of (a) (b) and that of $H_b(\boldsymbol{r}_c) - V_b(\boldsymbol{r}_c)/2$ versus w (c). Typical hydrogen bonds without covalency and typical hydrogen bonds with covalency are abbreviated as *t*-HB without cov. and *t*-HB with cov., respectively, whereas Cov-w and Cov-s stand for weak covalent bonds and strong covalent bonds, respectively.

Table SA1. Proposed definitions for the classification and characterization of interactions by the signs $H_b(\boldsymbol{r}_c)$ and $H_b(\boldsymbol{r}_c) - V_b(\boldsymbol{r}_c)/2$ and their first derivatives, together with the tentatively proposed definitions by the characteristic points on the plots of $H_b(\boldsymbol{r}_c)$ versus $H_b(\boldsymbol{r}_c) - V_b(\boldsymbol{r}_c)/2$. The tentatively proposed definitions are shown by italic. The requirements for the interactions are also shown.

ChP/Interaction	Requirements by $H_b(\boldsymbol{r}_c)$ and $V_b(\boldsymbol{r}_c)$	Requirements by $G_b(\boldsymbol{r}_c)$ and $V_b(\boldsymbol{r}_c)$
Origin	$H_b(\boldsymbol{r}_c) - V_b(\boldsymbol{r}_c)/2 = 0$; $H_b(\boldsymbol{r}_c) = 0$	$G_b(\boldsymbol{r}_c) = 0$; $V_b(\boldsymbol{r}_c) = 0$
vdW	$H_b(\boldsymbol{r}_c) > 0$; $dH_b(\boldsymbol{r}_c)/d(-r) > 0$	$G_b(\boldsymbol{r}_c) > -V_b(\boldsymbol{r}_c)$; $dG_b(\boldsymbol{r}_c)/d(-r) > -dV_b(\boldsymbol{r}_c)/d(-r)$
Borderline (BD-1)	$H_b(\boldsymbol{r}_c) > 0$; $dH_b(\boldsymbol{r}_c)/d(-r) = 0$	$G_b(\boldsymbol{r}_c) > -V_b(\boldsymbol{r}_c)$; $dG_b(\boldsymbol{r}_c)/d(-r) = -dV_b(\boldsymbol{r}_c)/d(-r)$
t-HB$_{\text{with no covalency}}$	$H_b(\boldsymbol{r}_c) > 0$; $dH_b(\boldsymbol{r}_c)/d(-r) < 0$	$G_b(\boldsymbol{r}_c) > -V_b(\boldsymbol{r}_c)$; $dG_b(\boldsymbol{r}_c) < -dV_b(\boldsymbol{r}_c)$
Borderline (x-intercept)	$H_b(\boldsymbol{r}_c) = 0$ ($\theta_p{}^a = 125°$)	$G_b(\boldsymbol{r}_c) = -V_b(\boldsymbol{r}_c)$ ($\theta_p{}^a = 125°$)
t-HB$_{\text{with covalency}}$	$H_b(\boldsymbol{r}_c) < 0$; (125° <) $\theta_p{}^a < 150°$	$G_b(\boldsymbol{r}_c) < -V_b(\boldsymbol{r}_c)$; (125° <) $\theta_p{}^b < 150°$
Borderline (Tentative)	$\theta_p{}^a = 150°$	$\theta_p{}^b = 150°$
CT-MC	$d(H_b(\boldsymbol{r}_c) - V_b(\boldsymbol{r}_c)/2)/d(-r) > 0$;	$dG_b(\boldsymbol{r}_c) > dV_b(\boldsymbol{r}_c)/2$;
	$150° < \theta_p{}^a < 180°$	$150° < \theta_p{}^a < 180°$
Borderline (BD-2)	$d(H_b(\boldsymbol{r}_c) - V_b(\boldsymbol{r}_c)/2)/d(-r) = 0$	$2dG_b(\boldsymbol{r}_c)/d(-r) = -dV_b(\boldsymbol{r}_c)/d(-r)$
	$(H_b(\boldsymbol{r}_c) - V_b(\boldsymbol{r}_c)/2 > 0$; $H_b(\boldsymbol{r}_c) < 0)$	$(-V_b(\boldsymbol{r}_c)/2 < G_b(\boldsymbol{r}_c) < -V_b(\boldsymbol{r}_c))$
CT-TBP with $X_3{}^-$	$d(H_b(\boldsymbol{r}_c) - V_b(\boldsymbol{r}_c)/2)/d(-r) < 0$	$2dG_b(\boldsymbol{r}_c)/d(-r) < -dV_b(\boldsymbol{r}_c)/d(-r)$
	$(H_b(\boldsymbol{r}_c) - V_b(\boldsymbol{r}_c)/2 > 0$; $H_b(\boldsymbol{r}_c) < 0)$	$(-V_b(\boldsymbol{r}_c)/2 < G_b(\boldsymbol{r}_c) < -V_b(\boldsymbol{r}_c))$
Borderline (y-intercept)	$H_b(\boldsymbol{r}_c) - V_b(\boldsymbol{r}_c)/2 = 0$ ($H_b(\boldsymbol{r}_c) < 0$)	$G_b(\boldsymbol{r}_c) = -V_b(\boldsymbol{r}_c)/2$ ($G_b(\boldsymbol{r}_c) < -V_b(\boldsymbol{r}_c)$)
Cov-w	$H_b(\boldsymbol{r}_c) - V_b(\boldsymbol{r}_c)/2 < 0$; $R^c < 0.15$ *au*	$G_b(\boldsymbol{r}_c) < -V_b(\boldsymbol{r}_c)/2$; $R^c < 0.15$ *au*
Borderline (Tentative)	$R^c = 0.15$ *au*	$R^d = 0.15$ *au*
Cov-s	$H_b(\boldsymbol{r}_c) - V_b(\boldsymbol{r}_c)/2 < 0$; $R^c > 0.15$ *au*	$G_b(\boldsymbol{r}_c) < -V_b(\boldsymbol{r}_c)/2$; $R^d > 0.15$ *au*

[a] $\theta_p = 90° - \tan^{-1}[dH_b(\boldsymbol{r}_c)/d(H_b(\boldsymbol{r}_c) - V_b(\boldsymbol{r}_c)/2)]$, $\theta_p = 125°$ is tentatively given for $\theta = 90°$, where θ is defined by $90° - \tan^{-1}[H_b(\boldsymbol{r}_c)/(H_b(\boldsymbol{r}_c) - V_b(\boldsymbol{r}_c)/2)]$ with $H_b(\boldsymbol{r}_c) = 0$. [b] $\theta_p = 90° - \tan^{-1}[d(G_b(\boldsymbol{r}_c) + V_b(\boldsymbol{r}_c))/d(G_b(\boldsymbol{r}_c) + V_b(\boldsymbol{r}_c)/2)]$, $\theta_p = 125°$ is tentatively given for $\theta = 90°$, where θ is defined by $90° - \tan^{-1}[(G_b(\boldsymbol{r}_c) + V_b(\boldsymbol{r}_c))/(G_b(\boldsymbol{r}_c) + V_b(\boldsymbol{r}_c)/2)]$ with $(G_b(\boldsymbol{r}_c) + V_b(\boldsymbol{r}_c)) = 0]$. [c] $R = [(H_b(\boldsymbol{r}_c) - V_b(\boldsymbol{r}_c)/2)^2 + (H_b(\boldsymbol{r}_c))^2]^{1/2}$. [d] $R = [(G_b(\boldsymbol{r}_c) + V_b(\boldsymbol{r}_c)/2)^2 + (G_b(\boldsymbol{r}_c) + V_b(\boldsymbol{r}_c))^2]^{1/2}$.

References

S1 (a) Bader, R.F.W. *Atoms in Molecules. A Quantum Theory*; Oxford University Press: Oxford, UK, 1990; (b) Matta, C.F.; Boyd, R.J. An Introduction to the Quantum Theory of Atoms in Molecules In The Quantum Theory of Atoms in Molecules: From Solid State to DNA and Drug Design; WILEY-VCH: Weinheim, Germany, 2007, Chapter 1.

S2 (a) Bader, R.F.W.; Slee, T.S.; Cremer, D.; Kraka, E. Description of conjugation and hyperconjugation in terms of electron distributions. *J. Am. Chem. Soc.* **1983**, *105*, 5061–5068; (b) Bader, R.F.W. A quantum theory of molecular structure and its applications. *Chem. Rev.* **1991**, *91*, 893–926; (c) Bader, R.F.W. A Bond Path: A Universal Indicator of Bonded Interactions. *J. Phys. Chem. A* **1998**, *102*, 7314–7323; (d) Biegler-König, F.; Bader, R.F.W.; Tang, T.H. Calculation of the average properties of atoms in molecules. *J. Comput. Chem.* **1982**, ***3***, 317–328; (e) Bader, R.F.W. Atoms in molecules. *Acc. Chem. Res.* **1985**, *18*, 9–15; (f) Tang, T.H.; Bader, R.F.W.; MacDougall, P. Structure and bonding in sulfur-nitrogen compounds. *Inorg. Chem.* **1985**, *24*, 2047–2053; (g) Biegler-König, F.; Schönbohm, J.; Bayles, D. AIM2000. *J. Comput. Chem.* **2001**, *22*, 545–559; (h) Biegler-König, F.; Schönbohm, J. Update of the AIM2000-Program for atoms in molecules. *J. Comput. Chem.* **2002**, *23*, 1489–1494.

S3 Nakanishi, W.; Nakamoto, T.; Hayashi, S.; Sasamori, T.; Tokitoh, N. Atoms-in-Molecules Analysis of Extended Hypervalent Five-Center, Six-Electron (5c–6e) C_2Z_2O Interactions at the 1,8,9-Positions of Anthraquinone and 9 -Methoxyanthracene Systems. *Chem. Eur. J.* **2007**, *13*, 255–268.

S4 (a) Nakanishi, W.; Hayashi, S.; Narahara, K. Polar Coordinate Representation of $H_b(\boldsymbol{r}_c)$ versus $(\hbar^2/8m)\nabla^2\rho_b(\boldsymbol{r}_c)$ at BCP in AIM Analysis: Classification and Evaluation of Weak to Strong Interactions. *J. Phys. Chem. A* **2009**, *113*, 10050–10057; (*b*) Nakanishi, W.; Hayashi, S. Atoms-in-Molecules Dual Parameter Analysis of Weak to Strong Interactions: Behaviors of Electronic Energy Densities versus Laplacian of Electron Densities at Bond Critical Points. *J. Phys. Chem. A* **2008**, *112*, 13593–13599.

S5 Nakanishi, W.; Hayashi, S. Atoms-in-Molecules Dual Functional Analysis of Weak to Strong Interactions. *Curr. Org. Chem.* **2010**, *14*, 181–197.

S6 (a) Nakanishi, W.; Hayashi, S. Dynamic Behaviors of Interactions: Application of Normal Coordinates of Internal Vibrations to AIM Dual Functional Analysis. *J. Phys. Chem. A* **2010**, *114*, 7423–7430; (b) Nakanishi, W.; Hayashi, S.; Matsuiwa, K.; Kitamoto, M. Applications of Normal Coordinates of Internal Vibrations to Generate Perturbed Structures: Dynamic Behavior of Weak to Strong Interactions Elucidated by Atoms-in-Molecules Dual Functional Analysis. *Bull. Chem. Soc. Jpn* **2012**, *85*, 1293–1305.

S7 Grimme, W.; Wortmann, J.; Frowein, D.; Lex, J.; Chen, G.; Gleiter, R. Laticyclic conjugated double bonds within the framework of oligocondensed bicyclo[2.2.2]octenes. *J. Chem. Soc., Perkin Trans. 2* **1998,** 1893–1900.

S8 Lin, C.-T.; Wang, N.-J.; Yeh, Y.-L.; Chou, T.-C. Synthesis, reactions and thermal properties of endo-5,12: endo-6,11-dietheno-5,5a,6,11,11a,12-hexahydronaphthacene. *Tetrahedron* **1995**, *51*, 2907–2928.

S9 Espinosa, E.; Alkorta, I.; Elguero, J.; Molins, E. From weak to strong interactions: A comprehensive analysis of the topological and energetic properties of the electron density distribution involving X–H···F–Y systems. *J. Chem. Phys.* **2002**, *117*, 5529–5542. See also Bianchi, R.; Gervasio, G.; Marabello, D. The experimental charge density in transition metal compounds. *C. R. Chim.* **2005**, *8*, 1392–1399.
